# c-Cbl mediates the degradation of tumorigenic nuclear β-catenin contributing to the heterogeneity in Wnt activity in colorectal tumors

**DOI:** 10.18632/oncotarget.12107

**Published:** 2016-09-20

**Authors:** Moshe Shashar, Jamaica Siwak, Umit Tapan, Shin Yin Lee, Rosana D. Meyer, Paige Parrack, Josenia Tan, Fatemeh Khatami, Jean Francis, Qing Zhao, Kevan Hartshorn, Vijaya B. Kolachalama, Nader Rahimi, Vipul Chitalia

**Affiliations:** ^1^ Department of Medicine, Boston University School of Medicine, Boston, MA, USA; ^2^ Department of Medicine, Hematology-Oncology Section, Boston University School of Medicine, Boston, MA, USA; ^3^ Department of Pathology, Boston University School of Medicine, Boston, MA, USA; ^4^ Whitaker Cardiovascular Institute, Boston University School of Medicine, Boston, MA, USA; ^5^ Cardiovascular Division, Brigham and Women's Hospital, Boston, MA, USA

**Keywords:** colorectal cancer, Cbl, Wnt

## Abstract

Despite the loss of *Adenomatous Polyposis Coli* (*APC*) in a majority of colorectal cancers (CRC), not all CRCs bear hallmarks of Wnt activation, such as nuclear β-catenin. This underscores the presence of other Wnt regulators that are important to define, given the pathogenic and prognostic roles of nuclear β-catenin in human CRC. Herein, we investigated the effect of Casitas B-lineage lymphoma (c-Cbl) on nuclear β-catenin, which is an oncoprotein upregulated in CRC due to loss-of-function *APC* or gain-of-function *CTNNB1* mutations. Despite mechanistic rationale and recent discoveries of c-Cbl's mutations in solid tumors, little is known about its functional importance in CRC. Our study in a cohort of human CRC patients demonstrated an inverse correlation between nuclear β-catenin and c-Cbl. Further investigation showed that the loss of c-Cbl activity significantly enhanced nuclear β-catenin and CRC tumor growth in cell culture and a mouse xenograft model. c-Cbl interacted with and downregulated β-catenin in a manner that was independent of *CTNNB1* or *APC* mutation status. This study demonstrates a previously unrecognized function of c-Cbl as a negative regulator of CRC.

## INTRODUCTION

In the United States, the lifetime risk for CRC is as high as 5% [1]. It is the second and the third most common cause of cancer death in men and women, respectively, and hence regulators of its pathogenesis are important to discern [1]. Aberrant hyperactive Wnt/β-catenin signaling is critical in CRC tumorigenesis [2, 3]. Loss-of-function *APC* mutations that are common in a majority of sporadic CRC and familial adenomatous polyposis coli (APC) patients are linked to Wnt hyperactivation. In a small subgroup of patients with wild-type *APC*, Wnt hyperactivation is due to a gain-of-function *CTNNB1 (β-catenin)* mutation. Both *APC* loss and the mutant *β-catenin* increase the transcriptionally ‘active’ β-catenin in the nucleus and are considered being ‘tumorigenic’ and the key initiators of CRC pathogenesis [2–4].

Despite widespread presence of *APC* mutations in CRC cases, the indicators of Wnt activity such as nuclear β-catenin remain heterogeneous [5, 6]. For example, though the biallelic loss of *APC* was present in almost all cases of familial *APC* and most CRC patients, nuclear β-catenin was rarely found in those polyps and in less than 50% of the adenocarcinoma cases [5–7]. While some of the CRC mice models such as colonic epithelial cell specific knock out CDX2P-NLS showed nuclear β-catenin [7], the others including rat PIRC and *APC^mcr^* zebrafish models displayed predominantly cytosolic/membrane β-catenin with little nuclear β-catenin [8, 9]. Forced expression of *KRAS* on the background of the loss of *APC* was required to induce nuclear β-catenin [10]. Taken together, accumulating evidence indicates the presence of other regulatory factors for nuclear β-catenin, which is clinically relevant given nuclear β-catenin is linked to an aggressive tumor phenotype, enhanced risk of metastasis and poor survival [11, 12].

Casitas B-lineage lymphoma (c-Cbl) is an E3 ubiquitin (UB) ligase containing a Src-homology-2 RING finger, which generally targets receptor tyrosine kinases (RTK) and other non-RTKs such as Src family kinases [13–15]. Unexpectedly, we uncovered β-catenin as a substrate of c-Cbl in Wnt-on phase in endothelial cells [16, 17]. CRC pathogenesis is driven by Wnt hyperactivation primarily due to active nuclear β-catenin due to *APC* loss or *CTNNB1* activating mutations, a state that is analogous to persistent Wnt-on phase. As c-Cbl is known to target β-catenin in Wnt-on phase [16, 17] and given the central role of active β-catenin in CRC tumorigenesis, we decided to examine the functional importance of c-Cbl in CRC.

c-Cbl mutations are well recognized in hematological malignancies [18–20] and additional mutations were recently identified in lung cancer, suggesting a possible role of c-Cbl in other solid tumors [21, 22]. Despite these findings and a mechanistic rationale, c-Cbl's role in CRC remains unexplored. In the present study, we examined the hypothesis that c-Cbl targets nuclear β-catenin to inhibit CRC tumor growth.

Using a color-based image segmentation technique, we quantified histological images derived from human CRC tumor tissues, and observed an inverse correlation between nuclear β-catenin and c-Cbl. Interfering with c-Cbl activity in CRC tumor cells and in an animal model enhanced the CRC tumor growth. Further mechanistic analysis revealed a physical interaction between c-Cbl and β-catenin and downregulation of β-catenin, which was independent of *APC* or *CTNNB1* mutation status and in a manner distinct from β-TrCP, a known E3 ubiquitin ligase of β-catenin [23]. Additionally, c-Cbl-mediated regulation of nuclear β-catenin was independent of its role in RTK signaling. The image quantification technique developed for this work has the potential for broad functionality and can be extended to estimate several aspects from histopathological images derived from heterogeneous tissues such as cancer.

## RESULTS

### c-Cbl expression inversely correlates with the nuclear β-catenin in CRC patients

To examine the role of c-Cbl in CRC, we investigated a possible relationship between c-Cbl and nuclear β-catenin in a cohort of 83 CRC biopsies of patients treated at Boston University School of Medicine from 2004-2014 (Table 1).

**Figure 1:**
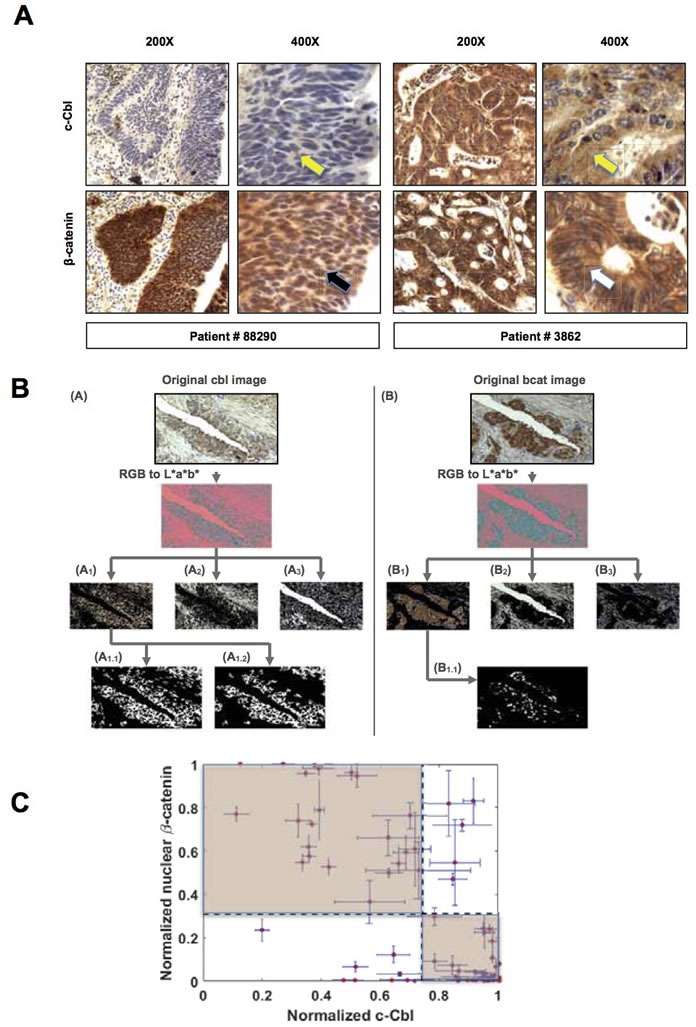
c-Cbl inversely correlates with nuclear β-catenin in human CRC **A.** Expression and localization of β-catenin and c-Cbl in two stage IV CRC patients showing a reverse expression pattern in β-catenin and c-Cbl. A set of consecutive slides stained for c-Cbl and β-catenin were examined at different magnifications (200X and 400X). Patient # 88290 showed a strong nuclear β-catenin (a black arrow), while c-Cbl expression was low (a yellow arrow). In contrast, patient # 3862 showed predominantly membrane and cytoplasmic β-catenin (a white arrow), and a high c-Cbl expression (a yellow arrow). **B.** Overview of the image-processing pipeline to estimate the amounts of nuclear β-catenin and c-Cbl. Two stages including RGB color image conversion to L*a*b space followed by segmentation of the color image into three sub-regions using a clustering algorithm are shown. Three basic sub-regions within each image: nuclei and its neighborhood, luminal area, and the interstitial space. (i) For the case of c-Cbl, the *k*-means algorithm (*k*=3) was used on each transformed color image in a*b* space and segmented into three clusters. From the three segmented clusters, the encapsulated cytoplasmic area within the entire image was identified. A size-based filtering operation was then performed on the identified cluster to eliminate all connected components that have fewer than 5000 pixels were removed from the identified cytoplasmic cluster. (ii) For nuclear β-catenin, the same clustering algorithm was used to first divide the transformed color image in a*b* space into three clusters. Each of the three clusters derived from the color image in a*b* space was divided into two sub-groups using the same *k*-means (*k*=2) clustering approach. The final outcome of this 2-tier clustering approach was 6 non-overlapping images. The surgical pathologists then identified the images with nuclear β-catenin. **C.** An inverse correlation between c-Cbl and nuclear β-catenin in CRC. A correlation between average normalized nuclear β-catenin and normalized c-Cbl was performed in 83 stage IV CRC patients. The dotted lines correspond to the averages of each parameter. Error bars = SEM. The highlighted areas represent groups with an inverse relationship between two parameters, and the divided cohort is depicted in 2×2 contingency table (Supplementary Table 1).

Two gastrointestinal surgical pathologists first conducted a pilot study in twenty CRC tumors to examine the expression of c-Cbl and β-catenin on a consecutive set of slides in a blinded fashion. A pre-validated β-catenin antibody was used [24]. c-Cbl antibody was validated using c-Cbl KO cells and with a blocking peptide corresponding to amino acids 892-906 of human c-Cbl (Supplementary Figures 1A-1C). The c-Cbl band was not observed in c-Cbl KO cells. c-Cbl signal reduced in a dose-dependent manner with the pre-incubation of increasing amount of blocking peptide with c-Cbl antibody. Using this validated antibody, immunohistochemistry studies revealed c-Cbl in the cytoplasm, (Figure 1A) and occasionally in the nucleus. β-catenin localized in the membrane, cytoplasm and nucleus. A trend towards an inverse relationship between c-Cbl and nuclear β-catenin was noted by the surgical pathologist using a semi-quantitative scale (low to high expression corresponding to 1+ to 3+ staining), a commonly used method for the assessment of protein expression. However, a quantitative method would be more suitable to uncover the relationship between two proteins by immunohistochemistry.

The image analysis technique developed for this study allowed us to estimate the amount of β-catenin and c-Cbl expression in the full cohort of 83 CRC patients. This strategy consisted of 4 mutually exclusive steps to estimate metrics of interest within the selected sub-regions of each image (Figure 1B). Multiple iterations involving fine-tuning of the clustering algorithm parameters along with the final review of the segmented images by the surgical pathologists allowed for the quantification of nuclear β-catenin and c-Cbl. These were normalized to the total tumor area in an image. The correlation was analyzed at both the patient-level (an average of all the images from each patient) and at the image-level (individual images). A significant negative correlation of average normalized c-Cbl and nuclear β-catenin was observed in 83 CRC patients (*p* < 0.0001, Spearman correlation −0.64) (Figure 1C), and also in 435 pairs of individual images (*p* < 0.001, Spearman correlation coefficient - 0.68) (Supplementary Figure 1D). The distribution of c-Cbl and nuclear β-catenin was further examined by dividing the cohort based on the averages of normalized nuclear β-catenin and c-Cbl (Figure 1C). Though a wide distribution of the relationship between c-Cbl and nuclear β-catenin was noted, 81% (69 out of 83) of patients exhibited an inverse relationship between them (Figure 1C highlighted areas and Supplementary Table 1). In concurrence with others [5–7], nuclear β-catenin was noted in only 37% (31 of 83) of CRC tumors. Wide error bars in both these parameters arise from the heterogeneity within the tumor. Taken together, our observations suggest an inverse correlation between c-Cbl expression and nuclear β-catenin in CRC patients.

**Table 1:** Baseline characteristics of CRC patients

**Median age (years)**	62
**% Male**	50.79
**Ethnic background (%)**	
White	*34.92*
African American	*33.33*
Hispanic	*20.63*
Other	*11.11*
**Right sided colon cancer(%)**	30.16
**Histologic Type (%)**	
Adenocarcinoma	80.95
Signet Ring	1.96
**Presented with metastatic disease (%)**	76.19
**Mean number of organs metastasized**	1.66

### Loss of *c-Cbl* augments CRC tumor growth

**Figure 2:**
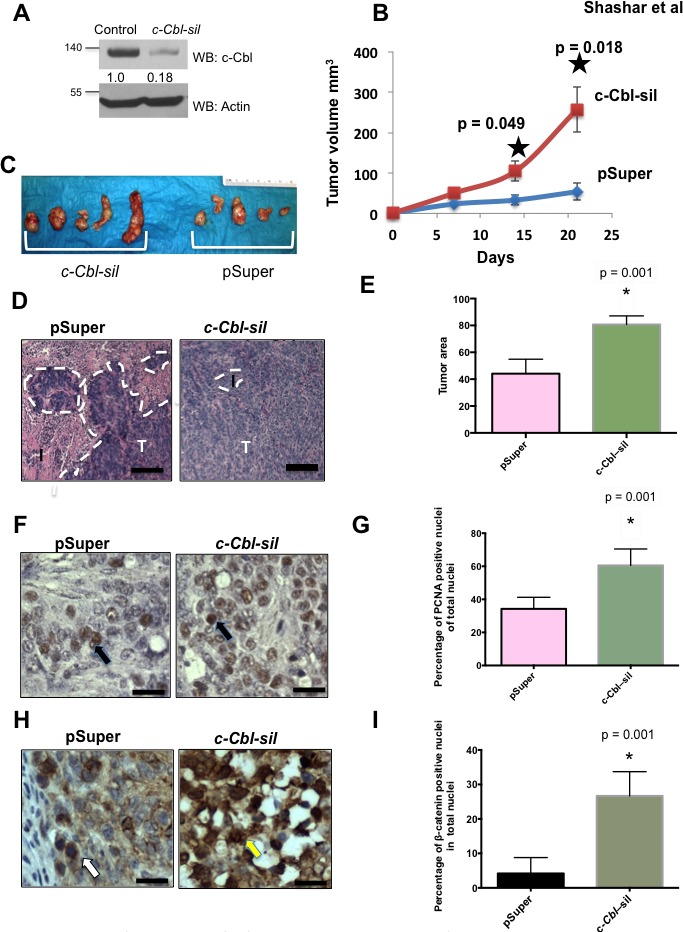
Loss of c-Cbl augments CRC tumor growth **A.** *c-Cbl* silenced (c*-Cbl*-*sil*) HT29 cells that were used for the xenograft model. HT 29 cells stably expressing control (pSuper) and *c-Cbl* shRNA(*c-Cbl*-sil) were lyzed and probed. The β-catenin band was normalized with actin for this figure and in all of the figures in this manuscript. A representative of the image from two repeat experiments is shown. **B.** *c-Cbl* silencing significantly increased the CRC tumor growth. HT29 cells (1×10^7^/animal) stably expressing control and silenced *c-Cbl* were mixed with Matrigel and injected subcutaneously into nude mice (*N* = 5). The growth of tumor cells was measured every seven days for three weeks. An average of tumor volume is shown. Error bars = SEM. **C.** Xenografts were removed on the day of harvest and dissected. The explants are shown. **D.** The tumor areas in *c-Cbl* silenced and control xenografts. Paraffin-embedded xenograft tumors were processed and stained for H & E. Ten slides per each xenograft were prepared (*N* = 5 xenografts from each group), and five random images were captured from each slide at 10X magnification. The area of the tumor, which includes adenocarcinoma without the interstitium or luminal regions, was measured using image quantification technique shown in Figure 1. In the first tier, this algorithm segments the area into three fundamental compartments including adenocarcinoma, interstitium, and lumen. The control tumors showed adenocarcinoma (the dotted line) interspersed with a small interstitial area, while in *c-Cbl* silenced xenografts showed predominant tumor area without interstitium. A representative from each group is shown, where I = interstitium and T = tumor, marked with the broken white line. Scale bar = 100 μM. **E.** The tumor area was significantly higher in *c-Cbl* silenced xenograft. A color-based segmentation pipeline was used to quantitate area of tumor in each slide as described above. Ten slides were prepared randomly from each xenograft (*N* = 5 for each group) and five random images per slides were analyzed. An average of tumor area is shown with error bars = SD. *p* values were calculated using Mann-Whitney U test. **F.** An increase in the proliferating cells in *c-Cbl* silenced CRC xenografts. Ten slides per each xenograft were processed and stained for PCNA, and counter-stained with H & E. Five random images were captured from each slide at 40X magnification and a representative from each group is shown. The black arrow indicates PCNA positive nuclei. Scale bar = 100 μM. **G.** PCNA positive nuclei were significantly higher in *c-Cbl* silenced xenografts. A color-based segmentation pipeline to quantitate PCNA positive nuclei was developed, as described above and expressed as percent of PCNA positive nuclei to the total nuclei. Ten slides were prepared randomly from each xenograft and five random images per slides were analyzed, and an average of number of PCNA positive nuclei is shown with error bars = SD. p values calculated using Mann-Whitney U test. **H.** An increase in the nuclear β-catenin in *c-Cbl* silenced xenograft. Xenograft slides processed and stained for β-catenin were analyzed as above. The yellow/white arrow indicates β-catenin positive nuclei. Scale bar = 100 μM. **I.** The cells with nuclear β-catenin were significantly higher in *c-Cbl* silenced xenografts. A color-based segmentation pipeline was used to quantitate β-catenin positive nuclei and normalized to a total number of nuclei. The percentage of β-catenin positive nuclei to the total number of nuclei is shown with the error bars = SD. p values calculated using Mann-Whitney U test.

Given the inverse correlation between c-Cbl and nuclear β-catenin in human tumors and the evidence that nuclear β-catenin regulates the growth of CRC [2–4], we examined the role of c-Cbl in CRC tumor growth in an *in vivo* xenograft model using *c-Cbl* silenced (*c-Cbl-sil*) HT-29 cells (Figure 2A). Compared to the controls, *c-Cbl* silenced mouse xenografts showed significantly increased tumor growth over 21 days (Figure 2B and 2C). The control xenografts showed a tumor (area demarcated by dashed white line) interspersed within the interstitium, while *c-Cbl* silenced xenografts had minimal interstitial area and significantly higher tumor area (Figure 2D and 2E). Next, we examined the proliferation of CRC cells using PCNA and the number of cells with nuclear β-catenin, both of which were normalized to a total number of nuclei. PCNA and β-catenin positive nuclei (Figure 2F-2I) were significantly higher in *c-Cbl* silenced xenografts indicating that reducing expression of c-Cbl in CRC tumor cells increases tumor cell proliferation. Taken together, these data indicate that expression of *c-Cbl* in CRC negatively regulates CRC tumor growth and downregulates nuclear β-catenin in CRC.

### c-Cbl interacts with and destabilizes different species of β-catenin in CRC

**Figure 3:**
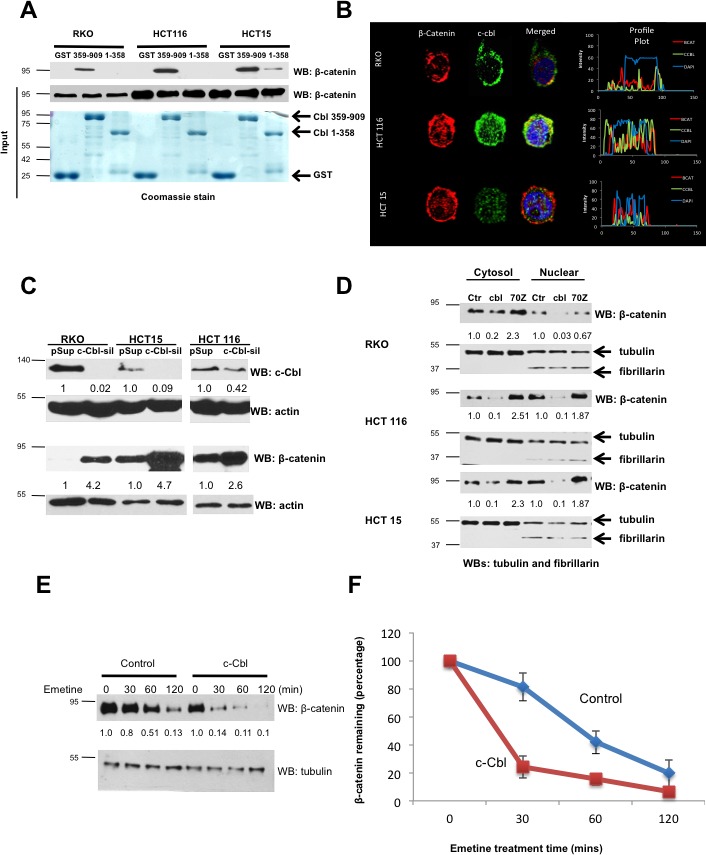
c-Cbl interacts and regulates tumorigenic β-catenin in CRC **A.** The binding of recombinant c-Cbl with different species of β-catenin. Recombinant GST or GST-tagged c-Cbl (1-358 and 359-909) were purified from E. coli and tethered to GST™ beads. The lysates from different CRC cell lines including RKO (wild-type β-catenin), HCT116 (phospho-resistant S33A mutant β-catenin) and HCT15 (active β-catenin in the *milieu* of loss of *APC*) were incubated for 4 hours with the beads, which then were washed with lysis buffer containing 0.2M NaCl. The eluents were probed using β-catenin antibody. Five percent of cell lysates and GST-tagged c-Cbl truncations stained using Coomassie dye are shown as inputs. Representative immunoblot from three experiments is shown. **B.** A co-localization of c-Cbl with wild-type, S33A mutant and active β-catenin in different CRC cell lines. CRC cells after 24 hours of seeding were fixed and stained for endogenous β-catenin and c-Cbl. DAPI was used for nuclear staining. Images obtained from the confocal microscope, were analyzed using ImageJ to generate the profile plots to demonstrate the distribution of the signal. **C.** *c-Cbl* silencing increased β-catenin in CRC cells. CRC cells were stably transduced with control (pSuper) or c-Cbl-silenced (*c-Cbl-sil*) shRNA and lyzed and probed using c-Cbl and β-catenin antibodies. Actin served as a loading control. A representative of three different experiments is shown. **D.** c-Cbl downregulated different species of β-catenin, while c-Cbl-70Z, an E3 UB ligase-deficient form of c-Cbl, served as a dominant negative. CRC cells stably expressing Flag-tagged c-Cbl (cbl) or c-Cbl-70Z or control (Ctr) were fractionated. Tubulin and fibrillarin served both as loading controls and the markers for the respective fractions. A representative of three different experiments is shown. **E.** *c-Cbl* destabilized active β-catenin in the *milieu* of *APC* loss. HCT15 cells stably transduced with the control and c-Cbl pretreated with 20μM emetine were harvested at the indicated time. A representative of three different experiments is shown. **F.** The stability of β-catenin was determined by the half-life, which is the time point corresponding to 50% of original amount of β-catenin after blocking the protein translation with emetine. β-catenin bands were normalized to tubulin and then to the amount of β-catenin at time zero. An average of three experiments is shown. Error bars = SD.

Our observations in human and mouse tumor xenograft model suggest a critical role for c-Cbl in downregulation of β-catenin. To further probe the role of c-Cbl in the regulation of β-catenin, we used a panel of CRC cell lines harboring different mutations in Wnt components (Supplementary Table 2) and examined c-Cbl interaction and its ability to regulate β-catenin. *In vitro* binding assays were performed using purified recombinant GST-tagged-c-Cbl C- or N- terminus truncations with the lysates from different CRC cell lines, namely S33A phospho-resistant mutant β-catenin (HCT116), active β-catenin in the *milieu* of *APC* loss (HCT15) and wild-type β-catenin (RKO cells) (Supplementary Table 2). The results demonstrated that the C-terminus of c-Cbl (c-Cbl 359-909) interacted with β-catenin in these CRC cell lines (Figure 3A). Furthermore, c-Cbl co-localized with different species of β-catenin in the nucleus and in the cytoplasm (Figure 3B). The data also demonstrate that the interaction of c-Cbl with β-catenin in CRC cells is independent of the status of *APC* or *β-catenin* mutations. These observations can be explained by the binding of c-Cbl to the Armadillo Repeat Motif (ARM) of β-catenin [16]. Both S33A mutant β-catenin and hypophosphorylated β-catenin due to the loss of *APC* have modified N-terminus of β-catenin but not the ARM motif. Hence, the binding to ARM motif endows c-Cbl the ability to interact with β-catenin independent of *APC* or *β-catenin* mutations.

Having established a physical interaction between c-Cbl and β-catenin in CRC cells, we next examined whether c-Cbl regulated the β-catenin in CRC cell lines. *c-Cbl* silenced CRC cells showed a 2.5-3.4 fold increase in total β-catenin and close to a 1.5-3 fold increase in nuclear β-catenin (Figure 3C and Supplementary Figure 2A). Unlike RKO and HCT15 cell lines, which had almost complete *c-Cbl* silencing, HCT116 showed silencing only up to 48%. Yet, this resulted in a 2.6-fold increase in β-catenin, a finding that raises a possibility of higher sensitivity of β-catenin in HCT116 cell line to c-Cbl, and may be explained by the type of mutation (S33A mutation) or other genomic heterogeneity in HCT116 cell line [25–28]. In an alternative approach, we over-expressed wild-type c-Cbl or dominant negative c-Cbl, c-Cbl-70Z, an E3 UB ligase-deficient dominant negative form of c-Cbl in CRC cell line. Overexpression of c-Cbl suppressed β-catenin and c-Cbl-70Z markedly increased β-catenin expression (Figure 3D).

We next examined whether c-Cbl destabilizes β-catenin. Half-life studies were conducted using emetine, a protein translational blocker, which generates a kinetic profile of protein degradation from samples harvested at different time points [16, 29]. c-Cbl overexpression significantly reduced the half-life of β-catenin from 48 minutes to 22 minutes (*p* = 0.012) (Figure 3E and 3F). On the other hand, *c-Cbl* silencing prolonged nuclear β-catenin half-life (average 30 minutes to 110 minutes, *p* = 0.01) (Supplementary Figures 2B and 2C). Also, *c-Cbl* silencing substantially reduced ubiquitination of active β-catenin in HCT15 cell line (Supplementary Figure 2D). Taken together, these data indicate that c-Cbl destabilizes and ubiquitinates different species of β-catenin in CRC.

β-Transducin Repeat Containing Protein (β-TrCP) is a well-characterized E3 UB ligase of β-catenin [23]. However, no comparative studies have been performed between β-TrCP and c-Cbl in CRC. We examined the effect of c-Cbl and β-TrCP on two discrete CRC cell lines harboring wild-type β-catenin (RKO) or S33A mutant β-catenin (HCT116). In RKO cells, *c-Cbl* and *β-TrCP* silencing each upregulated β-catenin and an additive effect was observed with the dual silencing of c-Cbl and β-TrCP (Supplementary Figure 2E). On the other hand, in HCT116 cells, *c-Cbl* silencing upregulated β-catenin, while *β-TrCP* silencing did not upregulate β-catenin. Co-silencing of *c-Cbl* in the *milieu* of *β-TrCP* silencing resulted in a 3-fold increase in β-catenin. Ubiquitination studies of β-catenin also corroborated with the above regulation. *c-Cbl* and *β-TrCP* silencing substantially reduced β-catenin ubiquitination in RKO cells, while only *c-Cbl* silencing reduced β-catenin ubiquitination in HCT116 cell line (Supplementary Figure 2F). These results suggest that c-Cbl exerts its effect on both the wild-type and S33A mutant β-catenin, while β-TrCP appear to regulate only wild-type β-catenin. These observations are consistent with the preferential binding of β-TrCP to the phosphorylated β-catenin, which is present in RKO cells but not in HCT116 cell lines harboring the phospho-resistant mutant form of β-catenin [23]. c-Cbl binds to the ARM region of β-catenin independent of its N-terminus phosphorylation or mutations in S33 residue, which explains continued c-Cbl regulation of β-catenin independent of *APC* or *CTNNB1* mutation status [16]. Taken together, these data indicate that c-Cbl destabilizes and ubiquitinates different species of β-catenin in a manner distinct from β-TrCP, which makes c-Cbl an important regulator of β-catenin in CRC.

### c-Cbl regulates Wnt target genes and CRC cell proliferation in CRC

**Figure 4:**
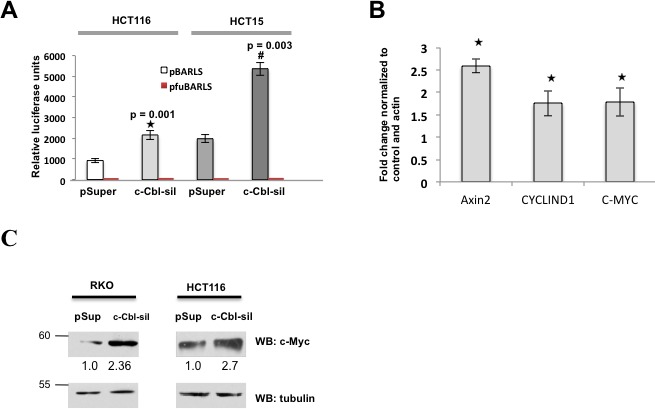
c-Cbl suppresses Wnt activity **A.** The *c-Cbl* silencing increased the Wnt activity in CRC cells with the S33A mutant β-catenin and the active β-catenin due to *APC* loss. The CRC cell lines were transfected with TCF-responsive promoter-reporter pBARLS and nonresponsive control reporter pfuBARLS tethered to a luciferase reporter (17). The Wnt activity was quantified by measuring relative firefly luciferase units normalized to protein concentration. An average of three independent experiments was shown. A Student's t-test was performed to determine the statistical significance. Error bars = SEM. **B.** The loss of *c-Cbl* activity upregulated Wnt target genes in CRC cells harboring the S33A mutant β-catenin. HCT116 cells stably expressing control or *c-Cbl* shRNA (c*-Cbl*-sil) were harvested within 24 hours of seeding. qRT-PCR reactions were run in duplicate for each sample and quantified using real-time PCR in triplicates for detecting *AXIN-2, C-MYC and CYCLIND1* mRNAs and the Ct values were generated. The values were normalized to the control and actin. An average of three independent experiments performed in duplicates is shown. Error bars = SEM. **C.** Doubling of c-Myc with *c-Cbl* silencing in CRC cell lines. The CRC silenced for *c-Cbl* were probed for c-Myc. Actin served as a loading control. A representative of two independent experiments is shown.

Considering a critical role of c-Cbl in the regulation of β-catenin, we posited that it is likely to modulate Wnt activity. *c-Cbl* silencing in CRC cells co-expressing β-catenin-mediated promoter-reporter constructs, showed a significantly higher Wnt activity (Figure 4A). β-catenin is a transcriptional coactivator, which induces transcription of several Wnt target genes including *AXIN2*, *MYC* and *CYCLIN D1* (Figure 4B). *c-Cbl* silenced cells showed close to 1.5-2 fold increase in the Wnt target genes. As *MYC* is considered to be a target gene regulated by Wnt/β-catenin, we also examined its protein levels, which doubled in *c-Cbl* silenced cells (Figure 4C), indicating that c-Cbl regulates Wnt activity and target genes in cells harboring either mutant *β-catenin* or active β-catenin due to *APC* loss.

**Figure 5:**
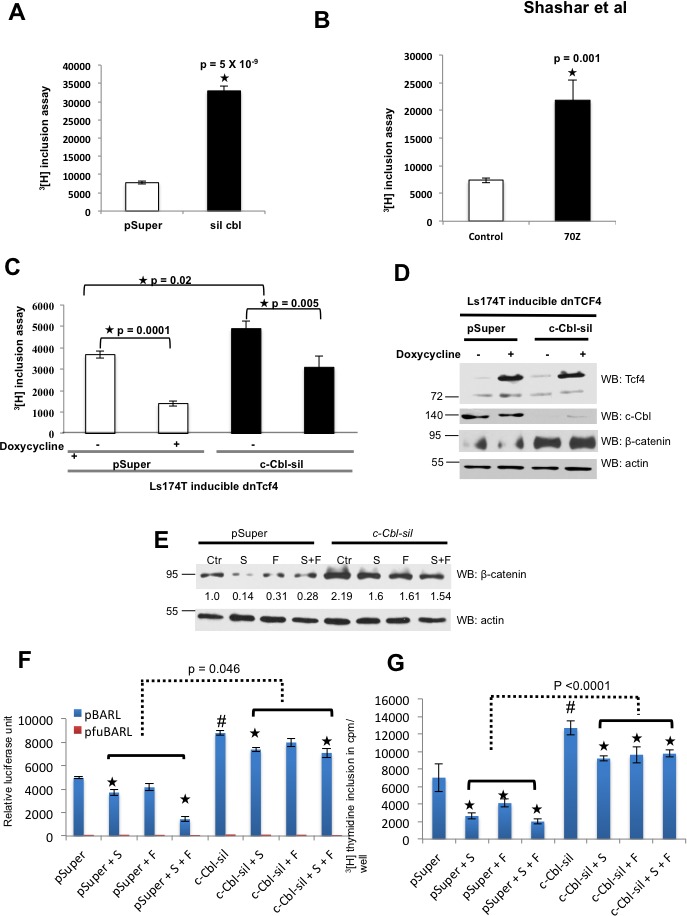
c-Cbl suppresses CRC cell proliferation independent of RTK-Wnt crosstalk **A.** *c-Cbl* silencing increased the proliferation of CRC cells harboring active β-catenin due to *APC* loss. HCT15 cells stably expressing control (pSuper) or *c-Cbl* shRNA (c*-Cbl*-sil) were serum starved for 24 hours and stimulated with 5% FBS. ^3^[H] incorporation assay was performed after 24 hours using The LabLogic 300SL Liquid Scintillation Counter. An average of six samples done in duplicates is shown. A Student's t-test was performed. Error bars = SEM. **B.** c-Cbl-70Z, an E3 UB ligase-deficient and a dominant negative form of c-Cbl increased the proliferation of CRC cells with S33A mutant β-catenin. HCT116 cells stably expressing control or *c-Cbl-70Z* construct were serum starved for 24 hours and stimulated with 5% FBS. ^3^[H] incorporation assay was performed after 24 hours. An average of six samples done in duplicates is shown. A Student's t-test was performed. Error bars = SEM. **C.** c-Cbl regulated the CRC proliferation in a TCF-4 dependent manner. Ls174T cells with inducible dominant negative (dnTCF4) were stably transduced with control (pSuper) or *c-Cbl* shRNA (c*-Cbl*-sil). The dnTCF4 was induced with doxycycline treatment for 5 days and the cells were processed as above. An average of six samples done in duplicates is shown. A Student's t-test was performed. The p values are shown for different comparisons. Compared to control, *c-Cbl* silenced cells showed a significant increase in the CRC proliferation. Induction of dnTC4 significantly reduced the Wnt activity in both the groups. Even in the dnTCF4 group, *c-Cbl* silenced cells showed a higher Wnt activity compared to the control cells (*p* =0.003). Error bars = SEM. **D.** The expression of dnTCF-4 and β-catenin are shown. The lysates from the above experiments were probed and the actin served as a loading control. **E.** c-Cbl continued β-catenin regulation even in the *milieu* of RTK suppression. LS174T cells stably expressing control (pSuper) or c-Cbl silenced shRNA (*c-Cbl-*sil) were treated with Sorafenib 25 μM, Foretinib 20 μM and same concentrations of Sorafenib + Foretinib for 24 hours based on IC50 (Supplementary Figures 4B, 4C). β-catenin bands were normalized to actin. A representative of three independent experiments is shown. A Student's t-test was performed to determine the statistical significance. Compared to pSuper, *c-Cbl* silenced cells had a 2-fold increase in β-catenin, *p* = 0.004. The group averages of treated cells were compared with untreated cells in pSuper and Silenced group using Wilcoxon rank sum test. A significant lower reduction was noted in treated c*-Cbl* silenced cells compared to the treated control cells (*p* = 0.0003). **F.** c-Cbl continued to regulate the Wnt activity in the *milieu* of RTK suppression. HT29 cells co-expressing TCF-responsive promoter-reporter pBARLS or nonresponsive control reporter pfuBARLS tethered to a luciferase reporter along with control (pSuper) or *c-Cbl* shRNA silenced cells were seeded in 96-well plates and serum starved overnight. The cells were stimulated with 5% FBS with Sorafenib or Foretinib or the same concentrations of Sorafenib + Foretinib, as described above. The cell lysates were subjected to luciferase assay using Promega Dual Luciferase Assay Kit^®^ according to the manufacturer's instructions and normalized to the protein content. An average of six independent samples is shown. A Student's t-test was performed to determine the statistical significance. Compared to the control cells (pSuper), the *c-Cbl* silenced (c*-Cbl*-sil) cells had 77% increased in the Wnt activity, (#) *p* = 0.00001. In the pSuper group, compared to the untreated cells, the cells treated with Sorafenib had (*) *p* = 0.02, Foretinib = 0.14 and Sorafenib + Foretinib treated cells had *p* = 0.0001. In the *c-Cbl* silenced group, compared to the untreated cells, the cells treated with Sorafenib (*) had *p* = 0.02, Foretinib *p* = 0.23 and Sorafenib + Foretinib *p* = 0.049. The group averages of treated cells were compared in pSuper and Silenced cells using Wilcoxon rank sum test (the dotted line, *p* = 0.046). Error bars = SEM. **G.** c-Cbl regulated the CRC proliferation even in the presence of RTK suppression. HT29 cells stably transduced with the control (pSuper) or *c-Cbl* shRNA (c*-Cbl*-sil) were seeded in a 96-well plate and serum starved. ^3^[H] thymidine incorporation assay (cpm/well) was performed after treating the cells with Sorafenib or Foretinib or Sorafenib + Foretinib as described above. An average of six samples is shown. A Student t-test was performed. Compared to the pSuper, the *c-Cbl* silenced cells showed a 44.69% increase in the cell proliferation, (#) *p* = 0.009. In the pSuper group, compared to the control, the cells treated with Sorafenib had (*) *p* = 0.02, Foretinib p = 0.109 and Sorafenib + Foretinib *p* = 0.011. In the Silenced group, compared to untreated cells, Sorafenib-treated cells had (*) *p* = 0.002, Foretinib *p* = 0.03 and Sorafenib + Foretinib p = 0.007. The group averages of treated cells were compared in pSuper and Silenced cells using Wilcoxon rank sum test (the dotted line, *p* <0.0001). Error bars = SEM.

Wnt target genes are potent regulators of CRC proliferation. In line with c-Cbl's Wnt suppressive effect, *c-Cbl* silencing (Figure 5A) and c-Cbl-70Z, a dominant negative c-Cbl, (Figure 5B and Supplementary Figures 3A, 3B) significantly increased ^3^[H] thymidine incorporation. These results corroborated with the significantly higher spheroid formation in *c-Cbl* silenced HT29 and HCT15 cell lines grown in suspension (Supplementary Figures 3C-3F). To further elucidate c-Cbl's regulation of β-catenin through β-catenin/TCF4 axis, we used a dominant negative TCF4 (dnTCF4) construct. As constitutively active dnTCF4 results in profound cell cycle arrest especially in those CRC cells with higher β-catenin [30], we used an inducible dnTCF4 (Figure 5C and 5D) available in LS174T cell line. Increased CRC cell proliferation induced by *c-Cbl* silencing was significantly abrogated with the induction of dnTCF4, indicating c-Cbl regulates CRC cell proliferation through Wnt-β-catenin-TCF4 axis.

c-Cbl downregulates multiple RTKs including EGFR, MET, and others [31], which in turn regulate Wnt signaling in different cancer types (RTK-Wnt crosstalk) (Supplementary Figure 4A) [32–36]. EGFR activates PI3/AKT pathway to inhibit GSK-3β and stabilize β-catenin [34]. EGFR activates PKM2 enzyme to bind to nuclear β-catenin and activate Wnt signaling [36]. Though an indirect regulation of β-catenin through c-Cbl-RTK-Wnt crosstalk is possible in CRC, together with the previous findings, the following observations support a direct regulation of β-catenin by c-Cbl. First, purified β-catenin directly binds to c-Cbl in an *in vitro* GST-pull down assay [16]. β-catenin preferentially binds to the C-terminus of c-Cbl, which is different from that of RTK binding (TKB/SH2 domain) (Figure 3B). Furthermore, a mutant c-Cbl construct (c-Cbl-G306E), which fails to regulate RTKs, can still downregulate β-catenin [16]. Lastly, c-Cbl regulates β-catenin in porcine aortic endothelial cells (PAECs), which do not express endogenous RTKs including, EGFR, FGFR, PDGFR, VEGFR and c-KIT [37, 38]. All these observations support an RTK-independent c-Cbl regulation of β-catenin.

To examine the RTK-independent and direct c-Cbl regulation of β-catenin, we suppressed RTK-Wnt crosstalk using a panel of RTK inhibitors with a hypothesis that the residual effect of c-Cbl on Wnt/β-catenin axis in the *milieu* of suppressed RTK can be attributed to the direct regulation of β-catenin by c-Cbl. A panel of RTK inhibitors was used at their IC_50_ levels including Foretinib (c-MET and VEGF receptor family inhibitor) [39]; Sorafenib (a broad-range inhibitor for Raf, FGFR-1, wild-type BRAF, mutant BRAF, VEGFR-2, PDGFR-β, PI3K and Flt3 kinases) [40]; and Gefitinib (predominantly an EGFR inhibitor) [41]. Their IC50 levels in CRC cells (Supplementary Figures 4A-4D) were in concurrence with others [39–41], and used to compare the percentage reductions in β-catenin expression, Wnt activity, and CRC cell proliferation between control and *c-Cbl* silenced cells.

In the control group, compared to untreated cells, the treated cells showed reduced β-catenin expression (mean +/− SD: 75.1 +/− 10.7). However, a significantly lower reduction in β-catenin expression was observed in treated *c-Cbl* silenced cells (22.52 +/− 8.4, Wilcoxon rank sum test *p* < 0.0003) (Figure 5E). A similar pattern was noted in Wnt activity (Figure 5F). In HT29 cells, both Sorafenib and a combination of Sorafenib with Foretinib significantly inhibited Wnt activity in the pSuper group (compared to the untreated cells, the percentage reduction in Wnt activity with the RTK inhibitors: mean +/− SD 37.46 +/− 27.60% relative luciferase unit - RLUs). Though the treatment with these agents also significantly inhibited Wnt activity in *c-Cbl* silenced group, the extent of Wnt inhibition was significantly compromised in *c-Cbl* silenced group (14.82 +/− 9.11% RLUs, Wilcoxon rank sum test *p* = 0.046). A similar pattern was observed in HCT116 treated with Sorafenib and Gefitinib (Supplementary Figure 4E).

The pattern of CRC proliferation corroborated with the effect on Wnt activity (Figure 5F and Supplementary Figure 4E). Suppression of CRC proliferation with RTK inhibitors was significantly higher in the control group (percentage reduction in proliferation with the treatment compared to the untreated cells; mean +/− SD: 54.17 +/−18.17%) compared to *c-Cbl* silenced cells (24.86 +/−11.39, *p* < 0.0001) (Figure 5G). Taken together, reduction in all three parameters - β-catenin expression, Wnt activity and CRC proliferation with RTK inhibitors were significantly lower in *c-Cbl* silenced cells compared to the control cells. Persistence of effect on Wnt/β-catenin axis in *c-Cbl* silenced cells with suppressed RTK signaling and RTK-Wnt crosstalk supports an additional RTK-independent direct component to c-Cbl regulation of β-catenin (Supplementary Figure 4A).

## DISCUSSION

Aberrant hyperactive Wnt signaling due to *APC or CTNNB1* mutations resulting in constitutively active β-catenin or S33A phospho-resistant β-catenin is the strongest causal link to human CRC [2–4]. Contrary to the expectation, nuclear β-catenin, which is a hallmark of overactive Wnt signaling, remains heterogeneous within CRC. This conundrum of stabilized β-catenin not being nuclear in CRC cells indicates a potential alternative and clinically relevant mechanism regulating the nuclear β-catenin [5, 6]. Our current work implicates c-Cbl as a negative regulator of different β-catenin species and tumor growth. Consistent with cell culture data, we also observed an inverse correlation between nuclear β-catenin and c-Cbl in a cohort of CRC patients. In addition to the indirect regulation through RTK-Wnt crosstalk, the current data uncovers a previously unrecognized RTK-independent component of c-Cbl's regulation of Wnt/β-catenin axis. The data also supports a distinct role of c-Cbl in CRC differentiating it from that of β-TrCP.

Owning to its frequent gain of function mutations in hematological malignancies, the role of c-Cbl is extensively studied in the context of hematological malignancies [18–20]. c-Cbl missense or small deletions predominantly in c-Cbl's RING finger and linker region contribute to myeloproliferative disorders, including atypical chronic myeloid leukemia, myelofibrosis juvenile myelomonocytic leukemia, myelodysplastic syndrome, etc. [18–20]. The mutant c-Cbl protein is thought to serve as a dominant negative for c-Cbl. Evidence also supports an additional gain-of-function component, where the mutant c-Cbl protein binds without degrading the RTKs inducing hyperactive tyrosine kinase signaling.

c-Cbl mutations were also recently described in lung cancer [21, 22], but their role remains largely unknown in other solid tumors like CRC. However, some inferences can be deduced from c-Cbl mutations in hematological malignancies, and lung cancers. Similar to the hematological malignancies, a loss of heterozygosity of c-Cbl was noted in lung cancer and its combinatorial effect with other mutations such as EGFR or Met was proposed as a mechanism of tumorigenesis [21, 22]. However, unlike hematological malignancies, the mutations in lung cancer patients predominated at the C-terminus and proline-rich region of c-Cbl and only a small proportion was found in its RING or linker domain [22]. Importantly, in contrast to the hematological malignancies, these mutations did not compromise the ubiquitination of EGFR, yet increased the survival, proliferation and motility of cancer cells, thereby suggesting other mediators for the tumor growth and metastatic enhancing properties of those mutants [22]. As β-catenin binds to C-terminus of c-Cbl (Figure 3A) and that the loss of binding increases its stability and Wnt activation [16, 17], these findings raise a possibility of β-catenin as a candidate effector of c-Cbl mutants.

These studies raise the question of why there is no increased incidence of solid tumors reported in patients with germline mutations of c-Cbl, despite the increase in the hematological malignancies. Higher expression of c-Cbl in the hematological compartment, differences in the types of c-Cbl mutations, the combinatorial influence of other oncogenic and tumor suppressor pathways, and the effect of the *milieu* on the tumorigenesis may provide possible explanations. We believe that these questions provide a strong rationale to examine c-Cbl in greater depth in solid tumors, and the current body of work is the first study probing the role of c-Cbl in CRC.

Our analysis also revealed differential roles of β-TrCP and c-Cbl in the regulation of β-catenin in CRC cells. In RKO cells harboring wild-type β-catenin susceptible to the phosphorylation by GSK-3β, silencing of individual E3 UB ligases increased β-catenin by a 2.5-fold but their combined silencing had higher effect (close to a 4-fold rise of β-catenin) (Supplementary Figure 2E). These findings may support the notion of separate pools of β-catenin targeted by these individual E3 UB ligases such that silencing of both results in an additive effect. It is likely that these pools are created due to binding of E3 UB ligases to different sites on β-catenin (β-TrCP to the phosphorylated N-terminus and c-Cbl to ARM region independent of the N-terminus phosphorylation) [16, 23]. These binding differences on β-catenin may also explain their differential regulation of β-catenin. *APC* loss and S33A mutant β-catenin respectively results in hypo-phosphorylated or phospho-resistant β-catenin species. Unlike β-TrCP, c-Cbl is able to bind and downregulate both these species. These observations highlight c-Cbl as a distinct and an effective E3 UB ligase for ‘tumorigenic’ β-catenin in CRC.

c-Cbl's effect on β-catenin can be confounded by RTK-Wnt crosstalk (Supplementary Figure 4A) [32–36]. This is particularly relevant in CRC, as activating mutations in several of these RTKs are found in 12-30% of CRC patients [42, 43]. To uncover the direct component, it would be ideal to completely inhibit RTKs and abolish RTK-Wnt crosstalk - an effect that is challenging to achieve given several RTKs active in CRC and regulated by c-Cbl. Therefore, a panel of broad-based kinase inhibitors was used. Significant persistence of β-catenin expression and Wnt activity despite RTK inhibition (Figure 5E-5F and Supplementary Figure 4E) indicated a substantial contribution of this direct c-Cbl-β-catenin axis in CRC.

Computer-based image processing for detailed quantitative histology is an evolving discipline in cancer research [44]. This analysis framework is important not only to elucidate the relationship of different proteins as illustrated above but also for the development of prognostic or efficacy biomarkers. Despite this pressing need, current methods remain largely semi-quantitative, where images of sub-regions of interest and signal intensity are graded on a discrete scale, and the data gets grouped into finite bins. Such estimates are not optimal for tissues with complex histopathologies, where finer details are sought for obtaining mechanistic insight. Moreover, these methods cannot fully capture the potential heterogeneity across an entire imaging slide. Manual interpretation is time consuming and not scalable in a high throughput manner. Although this technique was specifically customized for this work, the framework itself is generalizable and can have several other applications such as estimation of the tumor area and the number of PCNA positive nuclei, etc., in xenograft model (Figure 2D-2F), which indicates its broad functionality.

In line with the previous findings [5, 6], we observed nuclear β-catenin in 37% (31 of 83) of CRC patients and of which 83% (26 of 31) of patients had low c-Cbl (Supplementary Table 1). Ninety percent (43 of 48) of CRC with high c-Cbl showed low nuclear β-catenin. Both these observations support c-Cbl as a modifier of β-catenin in CRC. There can be several possible explanations for low c-Cbl in CRC such as methylation of the c-*Cbl* promoter or its post-translational modifications. It is also plausible that other upstream regulators may alter c-Cbl. For example, Lai et al demonstrated that the activation of MET kinase destabilizes c-Cbl through the proteasomal pathway [45], which can potentially reduce the regulation of β-catenin and activate the Wnt signaling. As activating mutations of *MET* receptor are present in 9% of CRC [46], they can potentially explain low c-Cbl levels in some of the CRC.

Low c-Cbl expression in CRC tumors is consistent with its potential tumor suppressor function. The presence of CRC with the high c-Cbl expression in the current series (Supplementary Table 1) may imply that c-Cbl serves as a tumor suppressor in a subset of CRC cases with lower c-Cbl expression. However, it is likely that the advanced stage of CRC (stage 4, Table 1) is associated with altered c-Cbl levels and that its levels may be lower in the earlier stage of CRC. Despite increase in its protein levels, it is also plausible that c-Cbl activity is inhibited by yet other unknown mechanisms, and this may explain the presence of nuclear β-catenin in 11% of CRC patients with high c-Cbl level. Future studies will probe into the regulators of c-Cbl and also its influence on disease phenotype including risk for metastasis and patient survival in CRC.

Overall, this study identifies c-Cbl as a unique E3 ubiquitin ligase of the tumorigenic forms of β-catenin that are central to CRC pathogenesis. By validating its relationship in a cohort of human CRC and within an animal model, this study identifies c-Cbl as a modifier for CRC pathogenesis and a contributor to the heterogeneity in Wnt activity in human CRC.

## MATERIALS AND METHODS

### Image quantification

We developed a customized, color-based image segmentation pipeline to estimate the amounts of nuclear β-Catenin, c-Cbl expression, PCNA positive nuclei for human CRC samples and xenografts. The pipeline consists of one or more of the following steps: basic image pre-processing, color-based segmentation, size-based morphology filtering and valid area computation of selected sub-regions within each image. All RGB color images were first converted to L*a*b* color space, and the two-dimensional a*b* space data was used for further processing. For the case of c-Cbl, each pre-processed image was first segmented into three clusters based on a distance-based clustering algorithm. This approach finds partitions in image data such that objects within each cluster are close to each other and far from objects in other clusters. From the three-segmented clusters, the surgical pathologists then identified the cluster that encapsulated the cytoplasmic area within the entire image. A size-based filtering operation was then performed on the identified cluster to eliminate all connected components that have fewer than a threshold level of pixel area. The size of the resulting sub-region was then estimated by computing the fraction of the non-zero pixels within the entire image, resulting in a measure of cytoplasmic c-Cbl content. For the case of β-catenin, the same clustering algorithm was used to first divide the full pre-processed image into three clusters. As the goal was to measure nuclear β-catenin, separating the nuclei that contained β-catenin from the other nuclei within the interstitial and other tissue regions were necessary. Therefore, each of the three clusters derived from the full pre-processed image was divided into two sub-groups using the same clustering approach. The surgical pathologists then identified the sub-grouped image(s) that contained nuclear β-catenin and the total fraction of the non-zero pixel area(s) from the image(s) was estimated as a measure of total nuclear β-catenin.

### Mouse xenograft model

Female *Nu/j* mice (5-6 weeks old; 16-20 g) were obtained from Jackson Laboratories. All mice were housed in a specific pathogen-free environment and all procedures were performed in a laminar airflow hood after obtaining IACUC permission from Boston University Medical Center. Stably *c-Cbl* silenced and control HT29 cell lines were maintained as adherent cultures in DMEM containing 10% FBS at 37°C in a humidified atmosphere of 5% CO_2_. Each mouse was inoculated subcutaneously at the right flank with 7.5 × 10^6^ cells in DMEM suspension with 30% Matrigel (Corning). Tumor size was measured in two dimensions using a caliper, and the tumor volume was expressed in mm^3^ using the formula: V=0.5*l* x 2*w*, where *l* and *w* are the length and width of the tumor, respectively. Tumors were grown for three weeks before harvesting.

### Immunohistochemistry

Xenograft tumor sections were fixed in 4% paraformaldehyde in PBS and then processed for paraffin embedding. Unstained 6 μm paraffin-embedded sections were deparaffinized and rehydrated through two changes of xylene and a graded alcohol series and then rinsed in dH_2_O. Antigens were retrieved by immersing sections in a pressure cooker containing boiling 1X Antigen Retrieval Citra Plus Solution (BioGenex) for 1 minute at 100°C. Sections were rinsed in dH_2_O and processed for immunostaining using EXPOSE Rabbit specific HRP/DAB detection IHC kit (Abcam) according to the manufacturer's protocol. Sections were counterstained with Harris Modified Hematoxylin (Fisher Scientific), dehydrated through a series of graded alcohol series and xylene, and mounted with EcoMount (BioCare Medical). Primary antibodies were β-catenin (H-102; 1:250; Santa Cruz Biotechnology) and PCNA (D3H8P; 1:2500; Cell Signaling Technology). c-Cbl antibody was validated using a blocking peptide. Increasing concentrations of c-Cbl blocking polypeptide (Santa Cruz Biotechnology Cat. # SC-170P) corresponding to amino acids 892-906 of human c-Cbl with sequence LREFVSISSPAHVAT were used. Increasing concentrations of blocking peptide were incubated along with 1:200 c-Cbl antibody (Santa Cruz Biotechnology Cat. # SC-170) for 30 minutes at room temperature before staining the paraffin-embedded tumor section of patients with colorectal cancer. The slides counterstained with H & E and images taken with the same setting of the microscope.

### Statistical analysis

Summary statistics are presented using the mean, median, and SD. Either a Student's t-test or Mann-Whitney U test was performed to compare the groups as appropriate. Spearman rank correlation was performed to examine association between two variables. For RTK inhibitors, a percent reduction for each value exposed to a drug relative to the mean value in the control was calculated and compared using a Wilcoxon rank sum test. Statistical significance was assessed at the *p* < 0.05.

## SUPPLEMENTARY MATERIALS FIGURES



## References

[1] Haggar FA, Boushey RP (2009). Colorectal cancer epidemiology: incidence, mortality, survival, and risk factors. Clin Colon Rectal Surg.

[2] Markowitz SD, Bertagnolli MM (2009). Molecular origins of cancer: Molecular basis of colorectal cancer. N Engl J Med.

[3] Fearon ER, Vogelstein B (1990). A genetic model for colorectal tumorigenesis. Cell.

[4] Behrens J (2000). Control of beta-catenin signaling in tumor development. Ann N Y Acad Sci.

[5] Anderson CB, Neufeld KL, White RL (2002). Subcellular distribution of Wnt pathway proteins in normal and neoplastic colon. Proc Natl Acad Sci U S A.

[6] Blaker H, Scholten M, Sutter C, Otto HF, Penzel R (2003). Somatic mutations in familial adenomatous polyps. Nuclear translocation of beta-catenin requires more than biallelic APC inactivation. Am J Clin Pathol.

[7] Hinoi T, Akyol A, Theisen BK, Ferguson DO, Greenson JK, Williams BO, Cho KR, Fearon ER (2007). Mouse model of colonic adenoma-carcinoma progression based on somatic Apc inactivation. Cancer Res.

[8] Feng Y, Sentani K, Wiese A, Sands E, Green M, Bommer GT, Cho KR, Fearon ER (2013). Sox9 induction, ectopic Paneth cells, and mitotic spindle axis defects in mouse colon adenomatous epithelium arising from conditional biallelic Apc inactivation. Am J Pathol.

[9] Amos-Landgraf JM, Kwong LN, Kendziorski CM, Reichelderfer M, Torrealba J, Weichert J, Haag JD, Chen KS, Waller JL, Gould MN, Dove WF (2007). A target-selected Apc-mutant rat kindred enhances the modeling of familial human colon cancer. Proc Natl Acad Sci U S A.

[10] Phelps RA, Chidester S, Dehghanizadeh S, Phelps J, Sandoval IT, Rai K, Broadbent T, Sarkar S, Burt RW, Jones DA (2009). A two-step model for colon adenoma initiation and progression caused by APC loss. Cell.

[11] Horst D, Reu S, Kriegl L, Engel J, Kirchner T, Jung A (2009). The intratumoral distribution of nuclear beta-catenin is a prognostic marker in colon cancer. Cancer.

[12] Chen Z, He X, Jia M, Liu Y, Qu D, Wu D, Wu P, Ni C, Zhang Z, Ye J, Xu J, Huang J (2013). beta-catenin overexpression in the nucleus predicts progress disease and unfavourable survival in colorectal cancer: a meta-analysis. PLoS One.

[13] Rao N, Miyake S, Reddi AL, Douillard P, Ghosh AK, Dodge IL, Zhou P, Fernandes ND, Band H (2002). Negative regulation of Lck by Cbl ubiquitin ligase. Proc Natl Acad Sci U S A.

[14] Mohapatra B, Ahmad G, Nadeau S, Zutshi N, An W, Scheffe S, Dong L, Feng D, Goetz B, Arya P, Bailey TA, Palermo N, Borgstahl GE (2013). Protein tyrosine kinase regulation by ubiquitination: critical roles of Cbl-family ubiquitin ligases. Biochim Biophys Acta.

[15] Singh AJ, Meyer RD, Navruzbekov G, Shelke R, Duan L, Band H, Leeman SE, Rahimi N (2007). A critical role for the E3-ligase activity of c-Cbl in VEGFR-2-mediated PLCgamma1 activation and angiogenesis. Proc Natl Acad Sci U S A.

[16] Chitalia V, Shivanna S, Martorell J, Meyer R, Edelman E, Rahimi N (2013). c-Cbl, a Ubiquitin E3 Ligase That Targets Active beta-Catenin: A NOVEL LAYER OF Wnt SIGNALING REGULATION. J Biol Chem.

[17] Shivanna S, Harrold I, Shashar M, Meyer R, Kiang C, Francis J, Zhao Q, Feng H, Edelman ER, Rahimi N, Chitalia VC (2015). The c-Cbl ubiquitin ligase regulates nuclear beta-catenin and angiogenesis by its tyrosine phosphorylation mediated through the Wnt signaling pathway. J Biol Chem.

[18] Shiba N, Kato M, Park MJ, Sanada M, Ito E, Fukushima K, Sako M, Arakawa H, Ogawa S, Hayashi Y (2010). CBL mutations in juvenile myelomonocytic leukemia and pediatric myelodysplastic syndrome. Leukemia.

[19] Niemeyer CM, Kang MW, Shin DH, Furlan I, Erlacher M, Bunin NJ, Bunda S, Finklestein JZ, Sakamoto KM, Gorr TA, Mehta P, Schmid I, Kropshofer G (2010). Germline CBL mutations cause developmental abnormalities and predispose to juvenile myelomonocytic leukemia. Nat Genet.

[20] Sanada M, Suzuki T, Shih LY, Otsu M, Kato M, Yamazaki S, Tamura A, Honda H, Sakata-Yanagimoto M, Kumano K, Oda H, Yamagata T, Takita J (2009). Gain-of-function of mutated C-CBL tumour suppressor in myeloid neoplasms. Nature.

[21] Imielinski M, Berger AH, Hammerman PS, Hernandez B, Pugh TJ, Hodis E, Cho J, Suh J, Capelletti M, Sivachenko A, Sougnez C, Auclair D, Lawrence MS (2012). Mapping the hallmarks of lung adenocarcinoma with massively parallel sequencing. Cell.

[22] Tan YH, Krishnaswamy S, Nandi S, Kanteti R, Vora S, Onel K, Hasina R, Lo FY, El-Hashani E, Cervantes G, Robinson M, Hsu HS, Kales SC (2010). CBL is frequently altered in lung cancers: its relationship to mutations in MET and EGFR tyrosine kinases. PLoS One.

[23] Winston JT, Strack P, Beer-Romero P, Chu CY, Elledge SJ, Harper JW (1999). The SCFbeta-TRCP-ubiquitin ligase complex associates specifically with phosphorylated destruction motifs in IkappaBalpha and beta-catenin and stimulates IkappaBalpha ubiquitination in vitro. Genes Dev.

[24] Feng Y, Sakamoto N, Wu R, Liu JY, Wiese A, Green ME, Green M, Akyol A, Roy BC, Zhai Y, Cho KR, Fearon ER (2015). Tissue-Specific Effects of Reduced beta-catenin Expression on Adenomatous Polyposis Coli Mutation-Instigated Tumorigenesis in Mouse Colon and Ovarian Epithelium. PLoS Genet.

[25] Rosin-Arbesfeld R, Cliffe A, Brabletz T, Bienz M (2003). Nuclear export of the APC tumour suppressor controls beta-catenin function in transcription. EMBO J.

[26] Gayet J, Zhou XP, Duval A, Rolland S, Hoang JM, Cottu P, Hamelin R (2001). Extensive characterization of genetic alterations in a series of human colorectal cancer cell lines. Oncogene.

[27] Feng Y, Bommer GT, Zhao J, Green M, Sands E, Zhai Y, Brown K, Burberry A, Cho KR, Fearon ER (2011). Mutant KRAS promotes hyperplasia and alters differentiation in the colon epithelium but does not expand the presumptive stem cell pool. Gastroenterology.

[28] Upadhyay G, Goessling W, North TE, Xavier R, Zon LI, Yajnik V (2008). Molecular association between beta-catenin degradation complex and Rac guanine exchange factor DOCK4 is essential for Wnt/beta-catenin signaling. Oncogene.

[29] Chitalia VC, Foy RL, Bachschmid MM, Zeng L, Panchenko MV, Zhou MI, Bharti A, Seldin DC, Lecker SH, Dominguez I, Cohen HT (2008). Jade-1 inhibits Wnt signalling by ubiquitylating beta-catenin and mediates Wnt pathway inhibition by pVHL. Nat Cell Biol.

[30] van de Wetering M, Sancho E, Verweij C, de Lau W, Oving I, Hurlstone A, van der Horn K, Batlle E, Coudreuse D, Haramis AP, Tjon-Pon-Fong M, Moerer P, van den Born M (2002). The beta-catenin/TCF-4 complex imposes a crypt progenitor phenotype on colorectal cancer cells. Cell.

[31] Schmidt MH, Dikic I (2005). The Cbl interactome and its functions. Nat Rev Mol Cell Biol.

[32] Krejci P, Aklian A, Kaucka M, Sevcikova E, Prochazkova J, Masek JK, Mikolka P, Pospisilova T, Spoustova T, Weis M, Paznekas WA, Wolf JH, Gutkind JS (2012). Receptor tyrosine kinases activate canonical WNT/beta-catenin signaling via MAP kinase/LRP6 pathway and direct beta-catenin phosphorylation. PLoS One.

[33] Piedra J, Miravet S, Castano J, Palmer HG, Heisterkamp N, Garcia de Herreros A, Dunach M (2003). p120 Catenin-associated Fer and Fyn tyrosine kinases regulate beta-catenin Tyr-142 phosphorylation and beta-catenin-alpha-catenin Interaction. Mol Cell Biol.

[34] Sharma M, Chuang WW, Sun Z (2002). Phosphatidylinositol 3-kinase/Akt stimulates androgen pathway through GSK3beta inhibition and nuclear beta-catenin accumulation. J Biol Chem.

[35] Ji H, Wang J, Nika H, Hawke D, Keezer S, Ge Q, Fang B, Fang X, Fang D, Litchfield DW, Aldape K, Lu Z (2009). EGF-induced ERK activation promotes CK2-mediated disassociation of alpha-Catenin from beta-Catenin and transactivation of beta-Catenin. Mol Cell.

[36] Yang W, Xia Y, Ji H, Zheng Y, Liang J, Huang W, Gao X, Aldape K, Lu Z (2011). Nuclear PKM2 regulates beta-catenin transactivation upon EGFR activation. Nature.

[37] Jiang X, Huang F, Marusyk A, Sorkin A (2003). Grb2 regulates internalization of EGF receptors through clathrin-coated pits. Mol Biol Cell.

[38] Meyer RD, Rahimi N (2003). Comparative structure-function analysis of VEGFR-1 and VEGFR-2: What have we learned from chimeric systems?. Ann N Y Acad Sci.

[39] Kataoka Y, Mukohara T, Tomioka H, Funakoshi Y, Kiyota N, Fujiwara Y, Yashiro M, Hirakawa K, Hirai M, Minami H (2012). Foretinib (GSK1363089), a multi-kinase inhibitor of MET and VEGFRs, inhibits growth of gastric cancer cell lines by blocking inter-receptor tyrosine kinase networks. Invest New Drugs.

[40] Wilhelm SM, Adnane L, Newell P, Villanueva A, Llovet JM, Lynch M (2008). Preclinical overview of sorafenib, a multikinase inhibitor that targets both Raf and VEGF and PDGF receptor tyrosine kinase signaling. Mol Cancer Ther.

[41] Feng YH, Tsao CJ, Wu CL, Chang JG, Lu PJ, Yeh KT, Shieh GS, Shiau AL, Lee JC (2010). Sprouty2 protein enhances the response to gefitinib through epidermal growth factor receptor in colon cancer cells. Cancer Sci.

[42] Laurent-Puig P, Lievre A, Blons H (2009). Mutations and response to epidermal growth factor receptor inhibitors. Clin Cancer Res.

[43] De Oliveira AT, Matos D, Logullo AF, SR DAS, Neto RA, Filho AL, Saad SS (2009). MET Is highly expressed in advanced stages of colorectal cancer and indicates worse prognosis and mortality. Anticancer Res.

[44] Beck AH, Sangoi AR, Leung S, Marinelli RJ, Nielsen TO, van de Vijver MJ, West RB, van de Rijn M, Koller D (2011). Systematic analysis of breast cancer morphology uncovers stromal features associated with survival. Sci Transl Med.

[45] Lai AZ, Durrant M, Zuo D, Ratcliffe CD, Park M (2012). Met kinase-dependent loss of the E3 ligase Cbl in gastric cancer. J Biol Chem.

[46] Lorenzato A, Olivero M, Patane S, Rosso E, Oliaro A, Comoglio PM, Di Renzo MF (2002). Novel somatic mutations of the MET oncogene in human carcinoma metastases activating cell motility and invasion. Cancer Res.

[47] Arthur D VS (2007). Proceedings of the eighteenth annual ACM-SIAM symposium on Discrete algorithms.

[48] Hastie T TR, Friedman J (2008). The elements of statistical learning.

[49] S L (2006). Least squares quantization in PCM. IEEE Trans Inf Theor.

