# Supplementary material for: c-Cbl mediates the degradation of tumorigenic nuclear β-catenin contributing to the heterogeneity in Wnt activity in colorectal tumors
Source: Oncotarget. 2016 Sep 20;7(44):71136–50. doi: 10.18632/oncotarget.12107 (PMC5342068; doi:10.18632/oncotarget.12107)
Supplement: Supplementary file 1 [file oncotarget-07-71136-s001.pdf]

c-Cbl mediates the degradation of tumorigenic nuclear β-catenin contributing to the heterogeneity in Wnt activity in colorectal tumors

Supplementary Material

Image quantification. We developed a customized color-based image segmentation pipeline to estimate the amounts of nuclear β-catenin, c-Cbl for human CRC samples and xenografts, and PCNA positive nuclei in xenografts (see **Figures** 1 and 2 and Supplementary Methods). A total of 83 CRC patients were included in the study and multiple histological images were taken per patient resulting in 393 x 2 images for quantifying c-Cbl content and nuclear β-catenin, respectively.

Image pre-processing:

Histological images of tissues were scanned at 20x using the Nikon TE2000 system, resulting in RGB color images. The size of each original image was 2560 x 1920 pixels. To ignore variations in brightness and quantify visual differences, each RBG image was first converted to L*a*b* space (47). This color space (also known as CIELAB or CIE L*a*b*) is derived from the CIE XYZ tristimulus values. Since one of the attributes of the L*a*b* model is device independence, the colors defined are independent of their nature of creation or the device they are displayed on. The L*a*b* space consists of a luminosity 'L*' or brightness layer, chromaticity layer 'a*' indicating where the color falls along the red-green axis, and chromaticity layer 'b*' indicating where the color falls along the blue-yellow axis. The a*b* space data of each image was then used for further processing.

Color-based segmentation:

Since the core of histological staining following by imaging is based on using color dyes to process, isolate and identify microscopic structures in a tissue, it was natural for us to exploit the color information to compute amounts of nuclear β-catenin and cytoplasmic c-Cbl. We used the *k*-means++ clustering algorithm to perform color-based segmentation, which is an iterative, data-partitioning algorithm that assigns *n* observations to exactly one of the *k* clusters defined

by centroids [47-49]. This algorithm treats each object as having a location in space and uses a heuristic to find centroid seeds for clustering. It finds partitions such that objects within each cluster are as close to each other as possible, and as far from objects in other clusters as possible. The approach requires that one specify the number of clusters to be partitioned and a distance metric to quantify how close two objects are to each other. For our case, we used a squared-Euclidean distance metric defined as

$d = (x-c)(x-c)'$ --- (1)

where x is the pixel of interest and c is the centroid, and d is the computed distance between the pixel and the centroid. Note that each centroid is the mean of the points in that cluster. For each pixel in the image, the clustering approach returns an index corresponding to a cluster. Using this index, one can separate objects by color. In order to ensure robustness, the clustering procedure using new initial cluster centroid positions was repeated ten times, and the solution with the lowest cluster sums of point-to-centroid distances was selected. Note that since the color information exists in the a*b* space, the objects derived using the clustering approach are pixels with a* and b* values.

Based on general observation of all the original images, the expert identified three basic sub-regions within each image: nuclei and its neighborhood, luminal area, and the interstitial space. This identification served as the basis for us to sub-divide each image into three clusters as the first step of color-based segmentation.

For the case of c-Cbl, the *k*-means algorithm (*k*=3) was used on each transformed color image in a*b* space and segmented into three clusters. From the three segmented clusters, the expert then identified the cluster that encapsulated the cytoplasmic area within the entire image. A size-based filtering operation was then performed on the identified cluster to eliminate all connected components that have fewer than a threshold level of pixel area. Several threshold values for the pixel areas were explored. After careful consideration, all connected components that have fewer than 5000 pixels were removed from the identified cytoplasmic cluster. The

resulting image was verified by the expert as the one comprising a filtered sub-region of c-Cbl content as exemplified by the colored staining within the cytosolic regions of each cell within the tissue.

For the case of β-catenin, the same clustering algorithm was used to first divide the transformed color image in a*b* space into three clusters. As the goal here was to estimate the amount of nuclear β-catenin, separating the nuclei that contained β-catenin from the other nuclei within the interstitial and other tissue regions were needed. Therefore, each of the three clusters derived from the color image in a*b* space was divided into two sub-groups using the same *k*-means (*k*=2) clustering approach. The final outcome of this 2-tier clustering approach was 6 non-overlapping images. The expert then identified the images with nuclear β-catenin. For many cases, one out of six images was identified to contain exclusive nuclear β-catenin but for some cases, there were at least 2 images that were identified to contain nuclear β-Catenin.

Valid area estimation of sub-regions per image:

For both c-Cbl and β-catenin, estimation of the size(s) of the resulting sub-region(s) was performed by computing the fraction of the non-zero pixels within the entire image(s). This resulted in a measure of cytoplasmic c-Cbl and nuclear β-catenin contents, respectively. When more than one image was identified (for nuclear β-catenin), a sum of the computed fractions of the images was considered as the valid area.

Cell culture. All CRC cell lines harboring different Wnt mutations including RKO, HCT15, HCT 116 and CaCo were grown in DMEM 10% FBS and 5% penicillin and streptomycin (**Supplementary table 1**). Ls174T cells obtained from Hans Clever lab (Hubrecht Institute, Germany) were grown with doxycycline 100 mg/ml for 5 days to induce the expression of dnTCF4, as described previously [30].

Chemicals. Sorafenib, Foretinib and Gefitinb all dissolved in DMSO were obtained from LC laboratories. Emetine was obtained from Sigma and MG132 from Calbiochem, EMD Millipore.

GST-tagged Protein Purification. GST purification of pGEX-2T c-Cbl (1–358), c-Cbl (359–

909), and β-catenin constructs was performed, as described previously [16].

GST pull down Assay. c-Cbl(1–358) or -(359–909) tethered to glutathione-SepharoseTM beads (GE Healthcare) were incubated with cell lysates of RKO,HCT15 HCT116 cells for 2 h at 4 °C in 50mM Tris-HCl, pH 7.6, 250 mM NaCl, 30 mM EDTA, 0.5% Triton X-100. Beads were extensively washed with the same buffer containing 300 mM NaCl and were boiled in Laemmli Buffer (Boston Bioproducts).

Immunoblotting and Immunoprecipitation. Cells were lysed in 50 mM Tris-HCl, pH 7.6, 150 mM NaCl, 30 mM EDTA, 0.5% Triton X-100 with complete protease inhibitor (Roche Applied Science). Immunoblotting and immunoprecipitation were performed, as described previously (5) and all the antibodies were from Cell Signaling (MA, USA), unless specified otherwise.

Immunofluorescence. Cells were grown in chamber slides (Lab-Tek) and fixed and processed as described previously. Alexa 488 goat anti-rabbit and Alexa 647 goat anti-mouse (Molecular Probes, Life Technologies) were used as secondary antibodies. ImageJ was used to generate the profile and the scatter plots, as described previously [16, 29].

Cellular Fractionation. Subcellular fractionation was performed using Dounce homogenization, as described previously [29].

Spheroid formation assay. 10,000 CRC cell were seeded in low adhesion plates (Corning ®) for 48 hours in complete growth medium and the spheroid colonies were counted in a blinded manner**.**

TCF/β-Catenin-responsive Luciferase Reporter Assay. The cells seeded in 6-well plates stably expressing c-Cbl or c-Cbl-70Z were cotransduced with lentiviral particles of pBARLS or pfuBARLS. After 48 h of transfection, luciferase assays were performed using the Dual-Luciferase Kit (Promega) and normalized using protein content determined by the Bradford assay (Bio-Rad).

Generation of Viral Particles. Retroviral constructs with c-Cbl overexpressing or *c-Cbl* or β-

TrCP shRNA constructs, as described previously [16, 17], were cotransfected into HEK293T cells along with packaging, envelope, and reverse transcriptase vectors using Lipofectamine 2000 (Life Technologies) per the manufacturer's instructions. Medium containing active viral particles collected after 48 h was centrifuged and stored at 80 °C. Lentiviral particles of TOP- and FOP-Flash were generated similarly by cotransfecting the lentiviral constructs with packaging, envelope, and reverse transcriptase vectors using Lipofectamine 2000 per the manufacturer's instructions. For viral transduction, the cells were seeded at 50–60% confluence. The cells were treated overnight with the medium containing active viral particles along with hexadimethrine bromide (Sigma), a cationic polymer, to increase the efficiency of infection. Puromycin (Sigma) selection was initiated after 24 h. The cells were harvested after four days to examine the effect on protein levels.

^{3}[H] Thymidine incorporation assay. 5,000 CRC cells seeded in 96 well plate were serum starved overnight. The cells were stimulated using DMEM medium containing 5% serum for 24 hours after which they were subjected to 1μCi of ^{3}[H] Thymidine overnight. The lyzed cells were counted for the radioactivity using the LabLogic 300SL Liquid Scintillation Counter.

Supplementary Table 1. Distribution of c-Cbl and nuclear β-catenin in CRC cohort

		**Average normalized c-Cbl**		
		**Low#**	**High#**	**Total**
**Average normalized nuclear β-catenin**	**Low***	**9**	**43**	**52**
	**High***	**26**	**5**	**31**
	**Total**	**35**	**48**	**83**

* = Average normalized nuclear β-catenin cut-offs is based on mean β-catenin (0.318) shown by the horizontal line in Figure 1C. For example, average normalized nuclear β-catenin less than 0.38 was considered low.

#= Average normalized c-Cbl cut-off is based on mean c-Cbl (0.74) shown by the vertical line in Figure 1C. For example, normalized average c-Cbl less than 0.74 was considered low.

Supplementary Table 2. A panel of CRC cell line with status of mutations in Wnt signaling and other components [25-28]

**Cell lines**	**Types of mutations in Wnt and RAS pathway genes**
RKO	Wild-type *APC*, *CTNNB1* and *EGFR*, wild-type *KRAS* but mutant *BRAF*
HCT116, LS174T	Gain-of-function *CTNNB1* mutation (Phosphorylation-resistant S33A β-catenin), wild-type *EGFR* and mutant *KRAS*
HCT15 and DLD1	Loss-of-function *APC* mutation resulting in active β-catenin, mutant *KRAS*
HT29	Loss-of-function *APC* mutation, wild-type *KRAS*, mutant *BRAF*

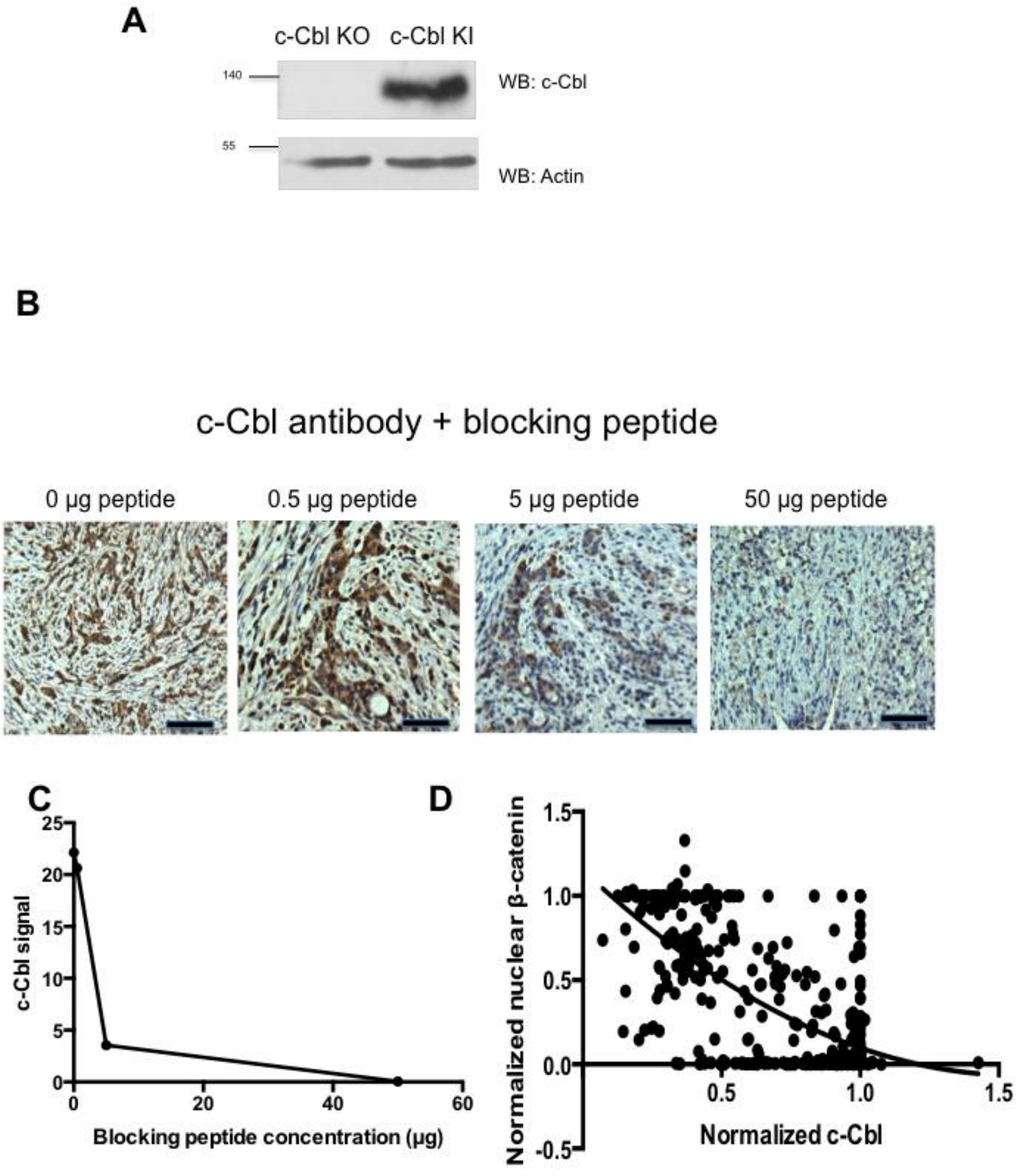

Supplementary Figure 1. Validation of c-Cbl antibody for IHC

A. c-Cbl antibody fails to detect c-Cbl in KO cells. Endothelial cells obtained from c-Cbl KO animals and cells where c-Cbl was knocked in (KI) were lyzed, and lysates were probed using 1:1000 rabbit polyclonal c-Cbl (C-15) antibody Santacruz Biotechnology cat. No.

SC-170. Actin served as a loading control. A c-Cbl band was not observed in c-Cbl KO cells. A representative from two independent experiments is shown.

B. c-Cbl signal is reduced with c-Cbl specific blocking peptide. Increasing concentration of peptide (Santacruz Biotechnology cat. No. SC-170P) corresponding to the amino acids 892-906 of human c-Cbl with sequence LREFVSISSPAHVAT was incubated with 1:100 c-Cbl antibody (SC-170) for 30 minutes at room temperature before staining the paraffin-embedded sections of a 53-year-old male stage IV CRC patient. The slides were counterstained with H & E and images were taken at the same setting of the microscope. A reduction of c-Cbl signal with increasing concentration of blocking peptide was noted.

C. The above images were analyzed using color based segmentation pipeline technique, where two-staged RGB color image conversion to L*a*b space was followed by the segmentation of the color image into three sub-regions using a clustering algorithm. The *k*-means algorithm (*k*=3) was used on each transformed color image in a*b* space and segmented into three clusters. From the three segmented clusters, the encapsulated area of interest within the entire image was identified by the surgical pathologists. c-Cbl staining was normalized to the total area of the tissue and then plotted against the concentration of blocking antibody. A reduction in c-Cbl signal is observed with increasing amount of blocking peptide.

D. An inverse correlation between normalized nuclear β-catenin and normalized c-Cbl at individual image level. 435 pairs of images of CRC biopsies stained for c-Cbl and β-catenin from 83 CRC patients were analyzed using a color-based segmentation pipeline technique and subjected to a correlation analysis. A Spearman correlation coefficient of -0.68 indicates a decrease in nuclear β-Catenin as c-Cbl increases and vice versa; and $p < 0.001$ indicates a highly significant association between these two variables.

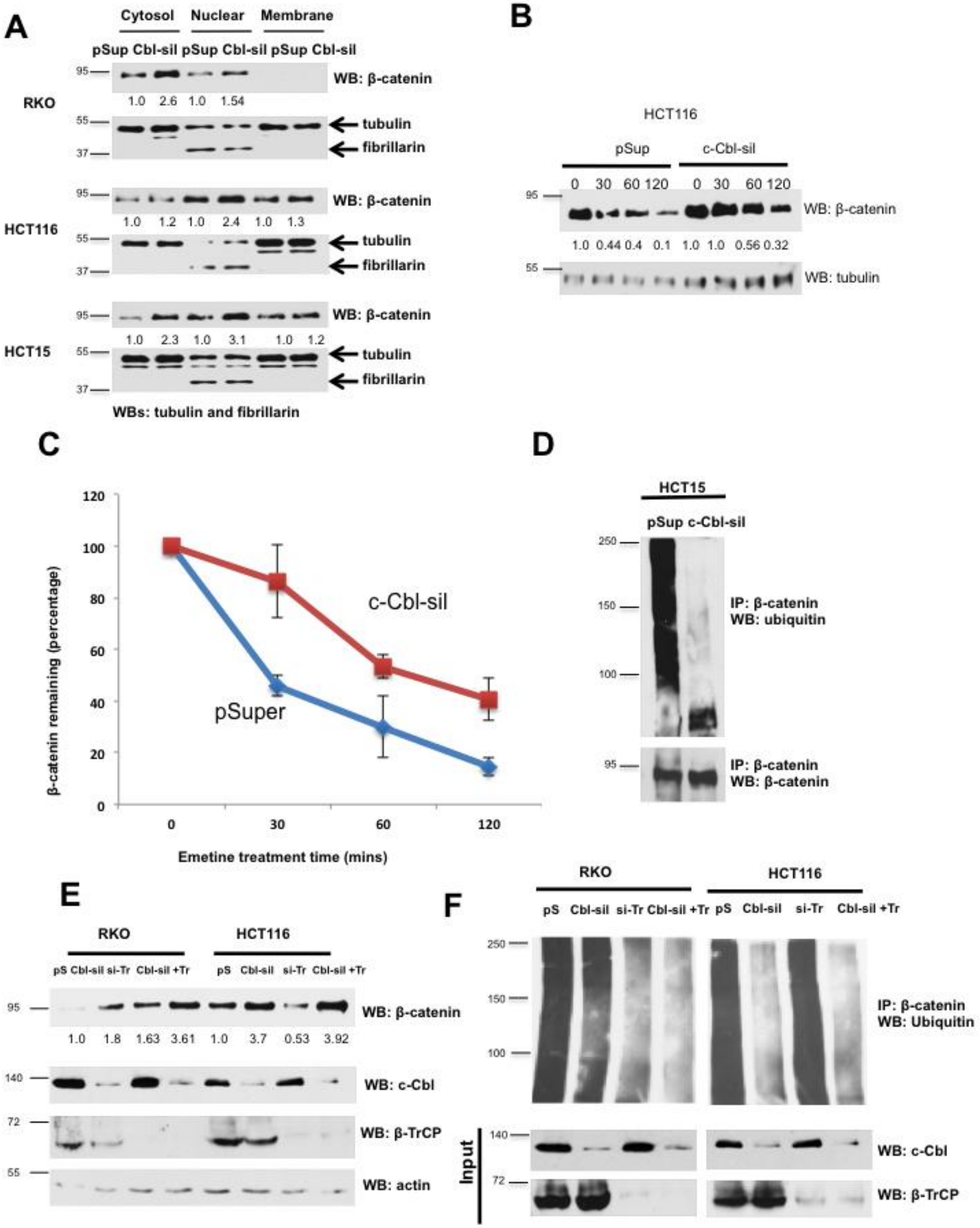
A
Cytosol Nuclear Membrane
pSup Cbl-sil pSup Cbl-sil pSup Cbl-sil
95
WB: β-catenin
RKO
1.0 2.6 1.0 1.54
55
tubulin
37
fibrillarin
95
WB: β-catenin
1.0 1.2 1.0 2.4 1.0 1.3
HCT116
55
tubulin
37
fibrillarin
95
WB: β-catenin
1.0 2.3 1.0 3.1 1.0 1.2
HCT15
55
tubulin
fibrillarin
37
WBs: tubulin and fibrillarin
B
HCT116
pSup c-Cbl-sil
0 30 60 120 0 30 60 120
95
WB: β-catenin
1.0 0.44 0.4 0.1 1.0 1.0 0.56 0.32
55
WB: tubulin
C
120
100
80
60
40
20
0
β-catenin remaining (percentage)
c-Cbl-sil
pSuper
0 30 60 120
Emetine treatment time (mins)
D
HCT15
pSup c-Cbl-sil
250
150
100
IP: β-catenin
WB: ubiquitin
95
IP: β-catenin
WB: β-catenin
E
RKO HCT116
pS Cbl-sil si-Tr Cbl-sil +Tr pS Cbl-sil si-Tr Cbl-sil +Tr
95
WB: β-catenin
1.0 1.8 1.63 3.61 1.0 3.7 0.53 3.92
140
WB: c-Cbl
72
WB: β-TrCP
55
WB: actin
F
RKO HCT116
pS Cbl-sil si-Tr Cbl-sil +Tr pS Cbl-sil si-Tr Cbl-sil +Tr
250
150
100
IP: β-catenin
WB: Ubiquitin
Input
140
WB: c-Cbl
72
WB: β-TrCP

Supplementary Figure 2. c-Cbl regulates cytosolic and nuclear fractions of β-catenin

A. *c-Cbl* silencing increases different species of β-catenin in CRC cells. CRC cells stably expressing control (pSup) or *c-Cbl* silencing (sil) retroviral constructs. The fractions were probed for β-catenin, and both tubulin and fibrillarin served as loading controls and as markers of cytosol and nuclear fractions respectively. A representative of three different experiments is shown.

B. Loss of *c-Cbl* activity increases the stability of phospho-resistant S33A mutant β-catenin. HCT116 cells stably transduced with the control (pSuper) and c-Cbl (sil-Cbl) shRNA were harvested after pretreatment with emetine. A representative of three different experiments is shown.

C. The stability of β-catenin was determined by the half-life, which is the time point corresponding to 50% of original amount of β-catenin after blocking the protein translation with emetine. β-catenin bands were normalized to tubulin and then to the amount of β-catenin at the time zero. An average of three experiments is shown. Error bars = SD

D. The loss of *c-Cbl* activity reduces the ubiquitination of active β-catenin in the *milieu* of *APC* loss. CRC stably expressing control (pSup) and *c-Cbl* silencing (sil) retroviral constructs were treated with 10 uM of MG132 for 16 hours and lyzed. The immunoprecipitated β-catenin was probed for ubiquitin. The stripped membrane was reprobed for β-catenin, which served as an input. A representative of three different experiments is shown.

E. A differential regulation of β-catenin by c-Cbl and β-TrCP. CRC cells stably expressing control shRNA (pS) or *c-Cbl* (si-c) or *β-TrCP* (si-Tr) or combined *c-Cbl* and *β-TrCP* (*si-c+Tr*) shRNAs were lyzed and probed. Actin served as a loading control. A representative of three different experiments is shown.

F. Ubiquitination of β-catenin by c-Cbl and β-TrCP. CRC cells stably expressing control

shRNA (pS) or *c-Cbl* (si-c) and/or *β-TrCP* (si-Tr) shRNAs pretreated with 10 μM MG132 for 16 hours were lyzed and immunoprecipitated using β-catenin antibodies and then probed with ubiquitin antibody. Five percent of lysates were probed as inputs. A representative of two independent experiments is shown.

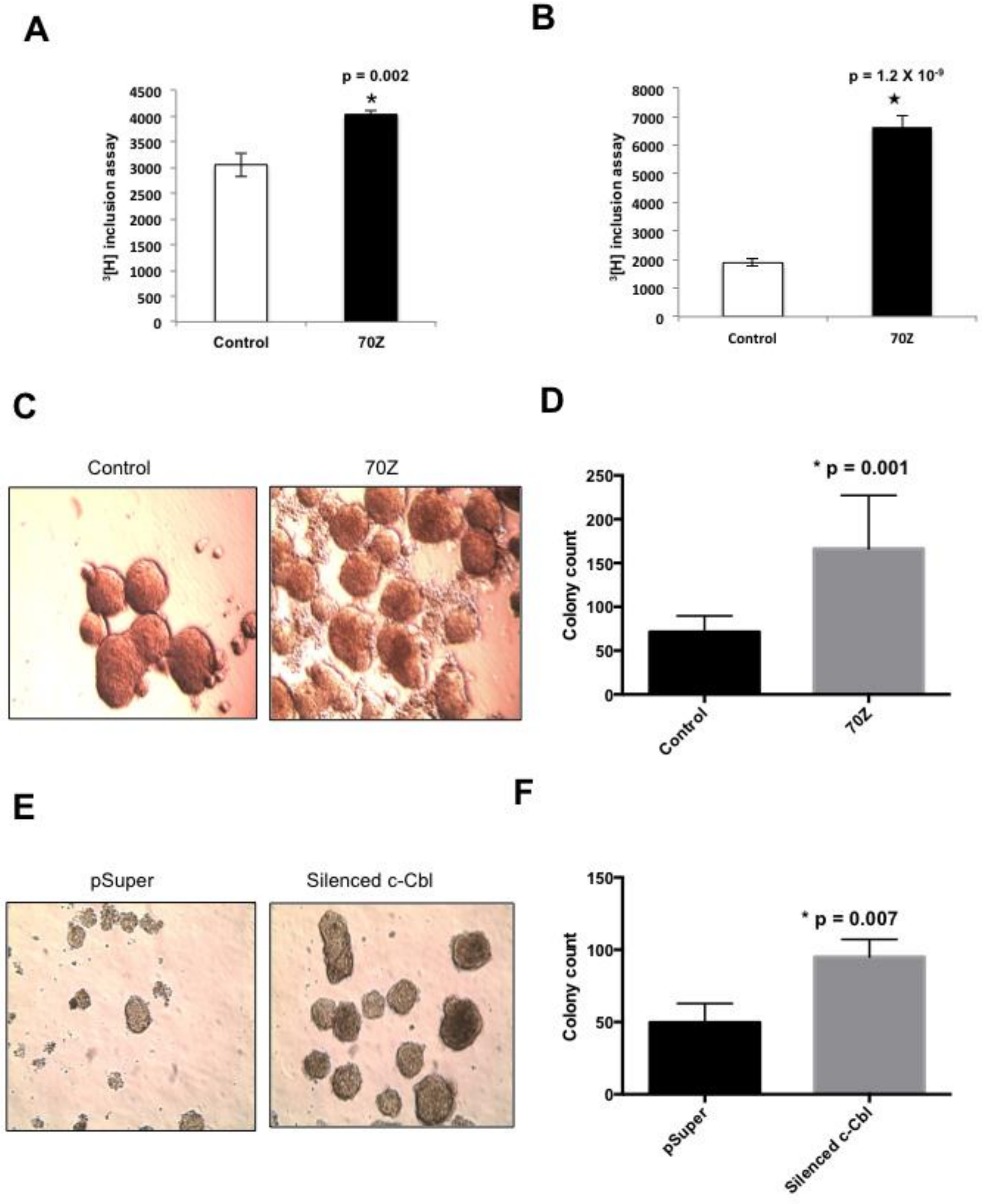

Supplementary Figure 3. c-Cbl regulates CRC proliferation and spheroid formation

A. c-Cbl-70Z, an E3 ligase-deficient and a dominant negative form of c-Cbl increases CRC cell proliferation harboring wild-type β-catenin. RKO stably expressing control or *c-Cbl-*

70Z constructs were serum starved for 24 hours and stimulated with 5% FBS. ^{3}[H] incorporation assay was performed after 24 h. An average of 6 samples done in duplicates is shown. A Student's t-test was performed. Error bars = SEM.

B. c-Cbl-70Z increases proliferation of CRC cells harboring S33A mutant β-catenin. HCT116 cells stably expressing control or *c-Cbl-70Z* constructs were serum starved and processed as above. An average of 6 samples done in duplicates is shown. A Student's t-test was performed. Error bars = SEM.

C. c-Cbl-70Z increases the spheroids formation in CRC cells harboring loss-of-activity *APC* mutation. HCT15 cells stably expressing control or c-Cbl-70Z were suspended in ultra-low adhesion plate (Corning) and imaged as mentioned above. Representative images from two separate experiments performed in triplicate are shown.

D. The number of spheroids was higher in cells expressing c-Cbl-70Z. The images were obtained as described above. The average number of spheroids is shown. Error bar = SEM. A Student's t-test was performed to compare the groups.

E. *c-Cbl* silenced cells produced a higher number of spheroids. HT29 cells stably expressing control (pSuper) or *c-Cbl* shRNA (silenced *c-Cbl*) were trypsinized, and 10,000 cells were suspended in ultra-low adhesion plate (Corning). Representative images from two independent experiments performed in triplicate are shown.

F. A higher number of spheroid formation in *c-Cbl* silenced CRC cells. Five random images were taken per well after 48 hours and the spheres counted in a blinded manner. The average number of spheroids is shown. A Student's t-test was performed to compare the group. Error bar = SEM.

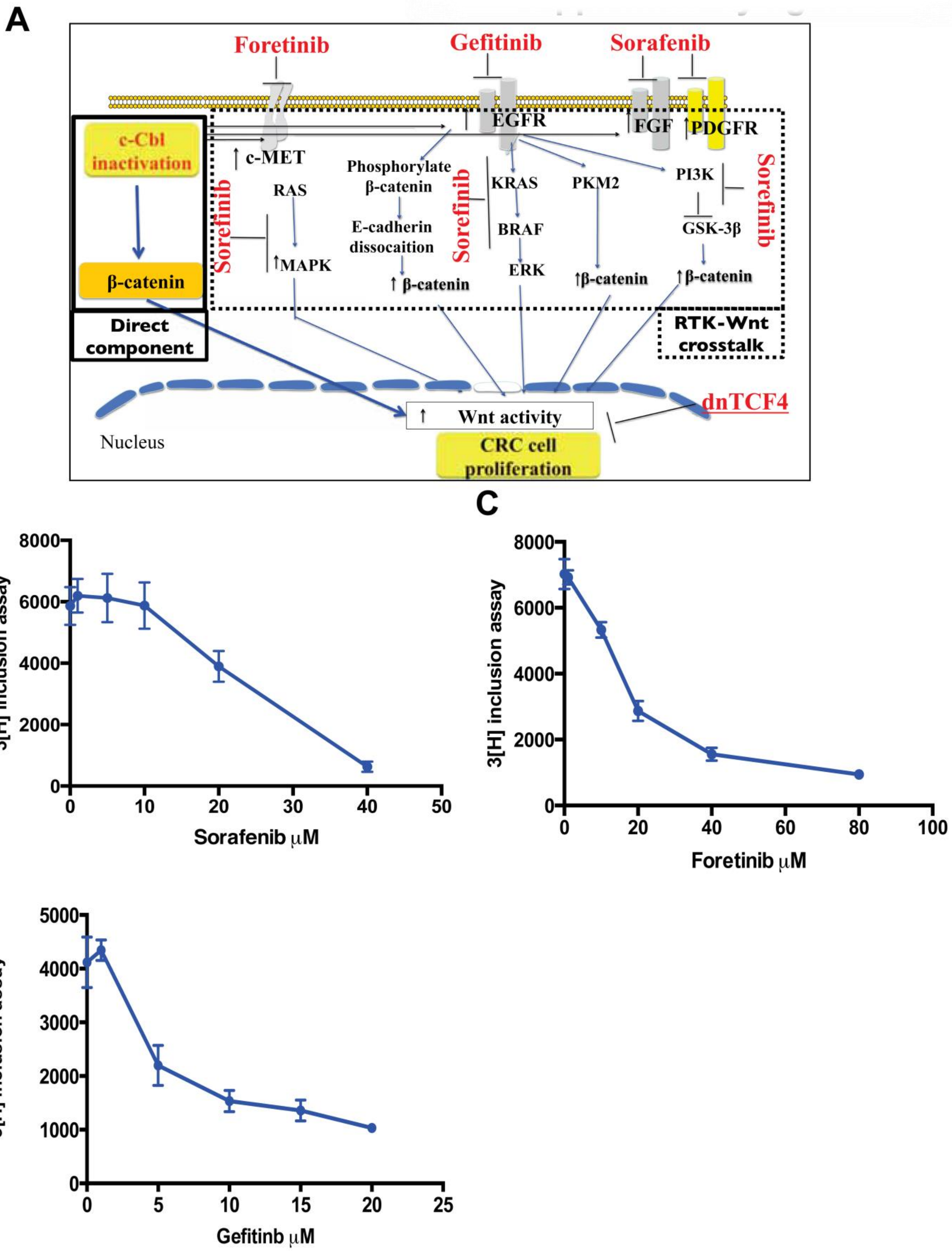
A
Foretinib
Gefitinib
Sorafenib
EGFR
FGF
PDGFR
c-Cbl inactivation
β-catenin
Direct component
c-MET
RAS
MAPK
Sorefinib
Phosphorylate β-catenin
E-cadherin dissocaition
β-catenin
Sorefinib
KRAS
BRAF
ERK
PKM2
β-catenin
PI3K
GSK-3β
β-catenin
Sorefinib
RTK-Wnt crosstalk
Nucleus
Wnt activity
dnTCF4
CRC cell proliferation
B
3[H] inclusion assay
Sorafenib μM
C
3[H] inclusion assay
Foretinib μM
D
3[H] inclusion assay
Gefitinb μM

E

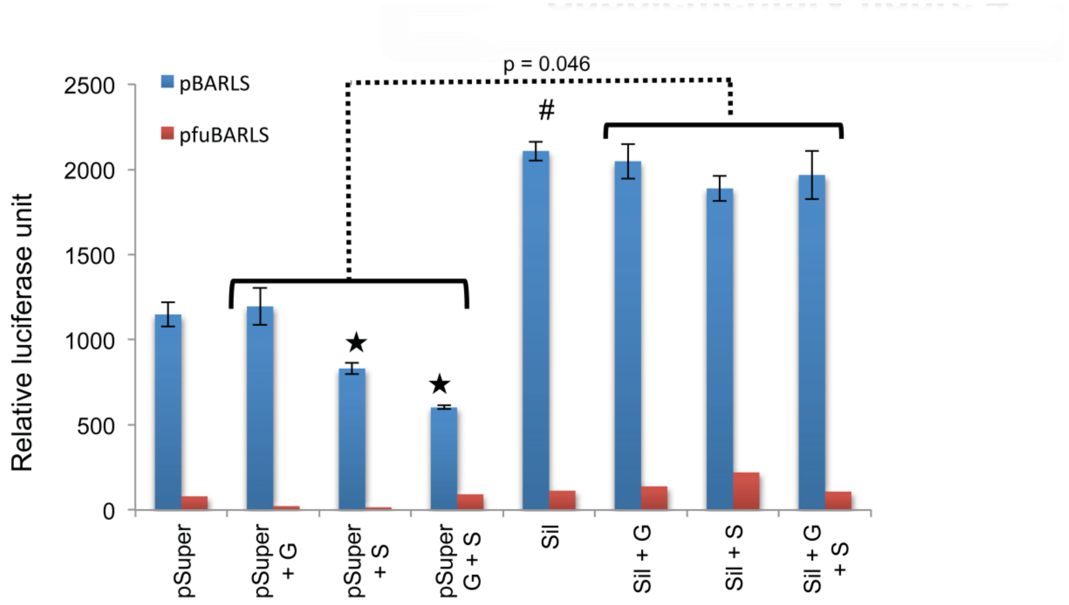

Supplementary Figure 4. Direct and RTK-mediated regulation of Wnt/ β-catenin by c-Cbl

A. c-Cbl downregulates multiple RTKs such as EGFR, FGF, PDGF, and c-MET. RTKs regulate Wnt signaling in different cancers and can potentially regulate Wnt signaling in CRC. In prostate cancer, EGFR activates PI3/AKT to inhibit GSK-3β and stabilize β-catenin [34]. In glioblastoma, EGF activates PKM2 enzyme to bind to nuclear β-catenin and activate Wnt signaling [36]. Also, EGFR directly binds and phosphorylates tyrosine residues on β-catenin to dissociate β-catenin from E-cadherin and thereby stabilizing β-catenin [32], and activating Ras/Raf/ERK pathway to induce Wnt signaling. Thus, c-Cbl can regulate Wnt/β-catenin through RTKs (RTK-Wnt crosstalk as show within the box with a dashed line) or its direct interaction (the box with a solid line). Kinase inhibitors used to interrogate the RTKs at different nodes of this network are shown.

B. IC_{50} of Sorafenib in HT29 cells. 10,000 HT29 cells seeded in 96 well plates were serum starved and stimulated with 5% FBS along with different concentrations of Sorafenib. After 24 hours, the cells were treated with 1μCi of ^{3}[H] Thymidine for 16 hours. The incorporation of radioactivity was measured using The LabLogic 300SL Liquid Scintillation Counter. The average of 6 samples is shown. Error bars = SEM. Sorafenib

concentration (25 µM) that reduced the proliferation of cells by 50% was considered as IC_{50} and used for subsequent assays.

C. IC_{50} of Foretinib. The IC_{50} of Foretinib was determined as above and found to be 20 µM for HT29 cells.

D. IC_{50} of Gefitinib was determined as above and was found to be 6 µM for HCT116 cells.

E. c-Cbl regulation of Wnt activity in CRC cells treated with kinase inhibitors. HCT116 cells co-expressing TCF-responsive promoter-reporter pBARLS or nonresponsive control reporter pfuBARLS tethered to the luciferase reporter along with control (pSuper) or c-Cbl shRNA (Sil) were seeded in 96 cells and serum starved overnight. The cells stimulated with 5% FBS with Sorafenib 25 µM, Gefitinib 7 µM and same concentrations of Sorafenib + Gefitinib for 24 hours depending on their IC_{50}, as determined above. The cell lysates were subjected to luciferase assay using Promega Dual Luciferase Assay Kit® and normalized to protein content. The average of 6 samples is shown. Student's t-test was performed to determine statistical significance. Compared to the control (pSuper), *c-Cbl* silenced (sil-Cbl) cells had 83% increased Wnt activity, (#) $p = 0.0004$. Compared to pSuper, Sorafenib and Gefitinib + Sorafenib-treated cells had (*) $p = 0.016$ and $p = 0.001$, respectively. Furthermore, a comparison was performed to examine if the percentage reduction of Wnt activity was different in the treated control and the *c-Cbl* silenced groups. Compared to the treated pSuper group (percentage inhibition of Wnt activity in treated mean +/- SD: 23.69+/- 24.11%), the extent of inhibition of Wnt activity was significantly lower in treated *c-Cbl* silenced group (mean +/- SD: 6.66 +/-8.42% RLUs, Wilcoxon rank sum test, $p = 0.046$). Error bars = SEM.
